# Bivariate genome-wide association analyses of the broad depression phenotype combined with major depressive disorder, bipolar disorder or schizophrenia reveal eight novel genetic loci for depression

**DOI:** 10.1038/s41380-018-0336-6

**Published:** 2019-01-09

**Authors:** Azmeraw T. Amare, Ahmad Vaez, Yi-Hsiang Hsu, Nese Direk, Zoha Kamali, David M. Howard, Andrew M. McIntosh, Henning Tiemeier, Ute Bültmann, Harold Snieder, Catharina A. Hartman

**Affiliations:** 1 Department of Epidemiology, University of Groningen, University Medical Center Groningen, Groningen, The Netherlands; 2 South Australian Health and Medical Research Institute (SAHMRI), Adelaide, SA Australia; 3 School of Medicine, University of Adelaide, Adelaide, SA Australia; 4 Division of Health Sciences, University of South Australia, Adelaide, SA Australia; 5 Department of Bioinformatics, Isfahan University of Medical Sciences, Isfahan, Iran; 6 HSL Institute for Aging Research, Harvard Medical School, Boston, MA USA; 7 Program for Quantitative Genomics, Harvard School of Public Health, Boston, MA USA; 8 Broad Institute of MIT and Harvard, Cambridge, MA USA; 9 Department of Epidemiology, Erasmus University Medical Center, Rotterdam, The Netherlands; 10 Department of Psychiatry, School of Medicine, Dokuz Eylul University, Izmir, Turkey; 11 Department of Genetics and Molecular Biology, School of Medicine, Isfahan University of Medical Sciences, Isfahan, Iran; 12 Division of Psychiatry, University of Edinburgh, Royal Edinburgh Hospital, Edinburgh, UK; 13 Centre for Cognitive Ageing and Cognitive Epidemiology, University of Edinburgh, Edinburgh, UK; 14 Department of Psychiatry, Erasmus University Medical Center, Rotterdam, The Netherlands; 15 Department of Health Sciences, Community and Occupational Medicine, University of Groningen, University Medical Center Groningen, Groningen, The Netherlands; 16 Interdisciplinary Center Psychopathology and Emotion Regulation, University of Groningen, University Medical Center Groningen, Groningen, The Netherlands

**Keywords:** Neuroscience, Depression, Genetics, Genetics, Depression

## Abstract

Although a genetic basis of depression has been well established in twin studies, identification of genome-wide significant loci has been difficult. We hypothesized that bivariate analyses of findings from a meta-analysis of genome-wide association studies (meta-GWASs) of the broad depression phenotype with those from meta-GWASs of self-reported and recurrent major depressive disorder (MDD), bipolar disorder and schizophrenia would enhance statistical power to identify novel genetic loci for depression. LD score regression analyses were first used to estimate the genetic correlations of broad depression with self-reported MDD, recurrent MDD, bipolar disorder and schizophrenia. Then, we performed four bivariate GWAS analyses. The genetic correlations (*r*_g_ ± SE) of broad depression with self-reported MDD, recurrent MDD, bipolar disorder and schizophrenia were 0.79 ± 0.07, 0.24 ± 0.08, 0.53 ± 0.09 and 0.57 ± 0.05, respectively. From a total of 20 independent genome-wide significant loci, 13 loci replicated of which 8 were novel for depression. These were *MUC21* for the broad depression phenotype with self-reported MDD and *ZNF804A*, *MIR3143*, *PSORS1C2*, *STK19*, *SPATA31D1*, *RTN1* and *TCF4* for the broad depression phenotype with schizophrenia. Post-GWAS functional analyses of these loci revealed their potential biological involvement in psychiatric disorders. Our results emphasize the genetic similarities among different psychiatric disorders and indicate that cross-disorder analyses may be the best way forward to accelerate gene finding for depression, or psychiatric disorders in general.

## Introduction

A depressive disorder is among the leading causes of morbidity and disability [1], and it is caused by a combination of genetic and environmental factors [2]. The genetic basis of depression is established by twin and family studies with heritability estimates ranging from 31 to 42% [3]. However, identification of genetic loci has turned out to be difficult, although more recent studies have started to be successful. Two loci were replicated based on 180,866 individuals in a meta-analysis combining two case–control studies on major depressive disorder (MDD) with UK Biobank data on a two-item measure of depressive symptoms [4]. A recent genome-wide association study (GWAS) that utilized online self-report data from 75,607 individuals reporting a clinical diagnosis or treatment of MDD and 231,747 controls, obtained through the personal genetics company 23andMe, Inc., identified 15 loci for MDD in a European ancestry population [5]. The CONVERGE consortium studied 5303 Han Chinese women with recurrent MDD and 5337 controls and identified two loci, near the *SIRT1* gene and in an intron of the *LHPP* gene [3]. Direk et al. [6] performed a joint analysis of the depressive symptoms GWAS meta-analysis (meta-GWAS) from the CHARGE consortium [7] and the MDD meta-GWAS from the Psychiatric Genomics Consortium (PGC) [8] involving 70,017 subjects. They discovered one genome-wide significant locus mapping to the *FHIT* gene for this broad depression phenotype [6]. Finally, the PGC MDD working group recently conducted a very large meta-GWAS in 130,664 MDD cases and 330,470 controls, and identified 44 independent loci [9].

Both MDD and symptoms of depression overlap with bipolar disorder and schizophrenia, and the diagnostic boundaries are sometimes difficult to define [10, 11]. For example, the so-called negative symptoms of schizophrenia are highly similar to several of the depression criteria including loss of interest, psychomotor retardation and low energy. Moreover, there is a significant overlap in the genetic risk of these psychiatric disorders. A substantial genetic correlation (*r*_g_ ± SE) was reported between MDD and schizophrenia (0.43 ± 0.06) and between MDD and bipolar disorder (0.47 ± 0.06) [12]. Also, a bioinformatic analysis of the PGC data demonstrated that MDD, bipolar disorder and schizophrenia share common biological pathways [13] and the latest PGC MDD GWAS showed significant bi-directional single-nucleotide polymorphism (SNP) effects in Mendelian randomization analyses between MDD and schizophrenia suggesting bi-directional causality [9].

Given these substantial phenotypic and genetic associations [12, 13], it can be argued that bipolar disorder and schizophrenia have a “depression” component that can be leveraged in bivariate analyses of depression with these other two psychiatric disorders to improve the power of gene discovery. The enhanced statistical power gained from combining data from related psychiatric disorders was recently illustrated by applying the bivariate genomic relatedness matrix restricted maximum likelihood (GREML) method implemented in GCTA software [12, 14, 15] followed by fitting multinomial logistic regression models that successfully identified genetic loci within the *CACNA1C*, *CACNB2* and *ITIH*3 genes, shared between the major psychiatric disorders [15]. However, these methods require the raw genotype and phenotype data of very large sample sizes [15, 16]. Recently proposed methods such as the bivariate linkage disequilibrium (LD) score regression [17] and bivariate meta-GWAS methods that rely on summary statistics from previous GWAS findings [18, 19] provide promising alternatives to estimate genetic correlations and subsequently identify shared genetic loci. These methods can be applied to (publicly) available data from large samples. In this study, we conducted four consecutive bivariate meta-analyses combining our recent meta-GWAS on the broad depression phenotype (depressive symptoms combined with MDD diagnosis) [6] with recent meta-GWASs on self-reported MDD [5], recurrent MDD [3], bipolar disorder [20] and schizophrenia [21] with the aim to identify novel genetic variants for depression. Genome-wide significant SNPs from the four bivariate GWAS analyses were replicated using UK Biobank results for broad depression [22].

## Materials and methods

We performed four consecutive bivariate analyses of the meta-GWASs of the broad depression phenotype [6], with online self-reported MDD [5], recurrent MDD [3], bipolar disorder [20] and schizophrenia [21]. The summary statistics of the GWAS results for self-reported MDD were provided by 23andMe on request and those for recurrent MDD, bipolar disorder and schizophrenia were available online (for details, see the Supplementary Materials and web resources).

### Statistical analyses

First, genetic correlations were estimated followed by a series of four bivariate meta-GWAS analyses. We also followed up our findings by annotating the genome-wide significant lead SNPs and conducting post-GWAS analysis as described below.

#### Step 1: Genetic correlation analyses

We applied the bivariate LD score regression method [17, 23] to estimate genetic correlations of the broad depression phenotype with self-reported MDD, bipolar disorder and schizophrenia, respectively. Genetic correlations summarize the genome-wide average effects of pleiotropy at shared loci [24]. This method relies on the LD score, provided together with the LDSC software and meta-GWAS summary statistics. The meta-GWAS summary statistics were obtained from the discovery stage meta-analyses of GWASs for the broad depression phenotype [6], self-reported MDD [5], bipolar disorder [20] and schizophrenia [21], while the LD scores were computed using the 1000 Genomes data of Europeans within a window size of ±1 Mb as described by Bulik-Sullivan et al. [17]. We used Popcorn for trans-ancestry estimation of the genetic correlation between our broad depression phenotype and recurrent MDD in the Chinese sample [25].

#### Step 2: Bivariate meta-analyses of genome-wide association analyses

A series of four bivariate meta-analyses were performed by combining meta-GWAS data of the broad depression phenotype with self-reported MDD, recurrent MDD, bipolar disorder and schizophrenia, respectively. We chose to do a series of bivariate analyses rather than a single multivariate analysis since the latter would unnecessarily focus the discovery of loci on the overlapping variance among all phenotypes studied. Analyzing combinations of two phenotypes at a time (i.e., the broad depression phenotype with (i) self-reported MDD (ii) recurrent MDD; (iii) bipolar disorder; and (iv) schizophrenia, respectively) should capture more shared variance thereby facilitating the identification and interpretation of novel loci for the broad depression phenotype. In this study, we applied a powerful bivariate GWAS analytical method using summary statistics from GWAS studies [26]. Our method takes into account the phenotypic correlations among dependent variables (phenotypes). This is in contrast to a classical (random-effect and fixed-effect) meta-analysis, in which the phenotypic correlations are not taken into account. We applied both the well-established O’Brien’s (OB) method and our own direct linear combination of dependent test statistics (dLC) approach [18, 19] as implemented in the C^++^ eLX package. The OB method and the dLC approach [18, 19] combine univariate meta-GWAS effect estimates (beta coefficients or *Z*-scores) of each SNP and yield a combined significance level based on the bivariate analysis. Power of these methods compares favorably to other popular multivariate methods such as that described by Porter and O'Reilly [27]. More details can be found elsewhere [18, 19, 26, 28].

In the bivariate meta-analyses, the full set of GWAS results was used to estimate variance–covariance matrices of test statistics. We combined the *Z*-scores of each SNP for the broad depression phenotype [6] with the *Z*-scores of: (i) self-reported MDD, (ii) recurrent MDD, (iii) bipolar disorder and (iv) schizophrenia. The analyses generate two test statistics and associated *p* values, one for the OB method and the other for the dLC method. Statistical significance of the bivariate association was determined based on the smaller of the two *p* values. The results were considered genome-wide significant if: (i) the *p* value for the bivariate analysis reached genome-wide significance (*p* < 5 × 10^–8^); (ii) the bivariate *p* value was one order of magnitude smaller than the univariate p-values of both analyzed phenotypes; (iii) the univariate meta-GWAS effects were at least nominally significant with a *p* < 0.001; and (iv) were in the same direction. For each bivariate GWAS analysis, only one independent lead SNP per locus was reported. Nearby SNPs in LD (*r*^2^ > 0.10) with the lead SNP were considered dependent and belonging to the same locus.

#### Step 3: Bivariate negative control GWAS analyses

See Supplementary Materials.

#### Step 4: Replication of genome-wide significant loci in UK Biobank

Genome-wide significant SNPs from the four bivariate GWAS analyses were replicated using UK Biobank results for broad depression [22] and subsequently meta-analyzed by the dLC approach using the genome-wide summary statistics [26]. SNPs were considered replicated if: (i) 1-sided *p* < 0.05, (ii) the effect was directionally consistent and (iii) the combined discovery and replication meta-analysis *p* value was more significant than its corresponding bivariate GWAS. Additionally, we performed a lookup of our genome-wide significant SNPs for probable MDD and ICD-coded MDD [22].

#### Step 5: Post-GWAS analyses

We used the post-GWAS pipeline reported by Vaez et al. [29] to annotate findings of our replicated SNPs in combination with those of established depression loci from the literature [4–6, 9]. The analyses approach includes in silico sequencing, in silico lookup of associations with other phenotypes in the GWAS catalog [30], expression quantitative trait loci analysis and functional network and enrichment analysis. Additionally, DEPICT [31] was used to prioritize genes, identify gene sets and evaluate tissue enrichments using results of the four bivariate GWAS with *p* < 1 × 10^–5^ as input (see Supplementary Materials for details).

## Results

### Broad depression phenotype with self-reported MDD

**Fig. 1:**
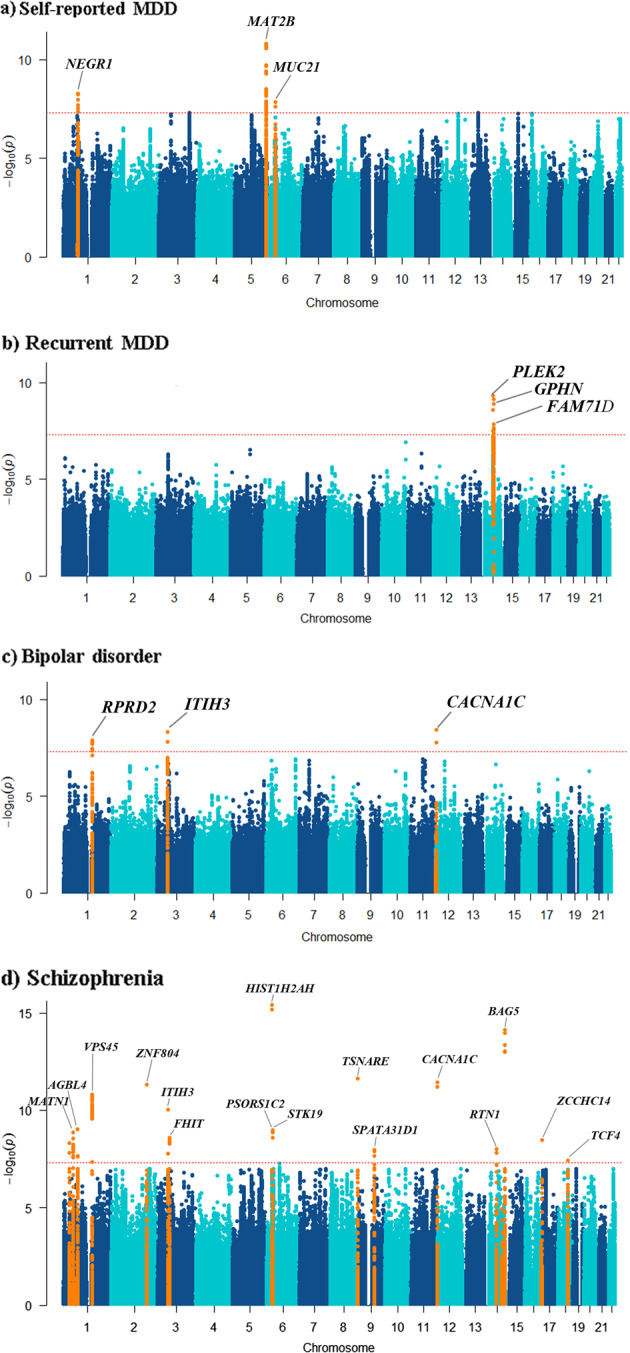
Manhattan plots showing the result of bivariate analysis between the broad depression phenotype and **a** self-reported MDD; **b** recurrent MDD; **c** bipolar disorder; and **d** schizophrenia, highlighting the loci that showed genome-wide significance (orange). The −log10 (bivariate *p* value) is plotted against the physical position of each SNP on each chromosome. The threshold for genome-wide significance (bivariate *p* value < 5 × 10^–8^) is indicated by the red dotted horizontal line

The genetic correlation (*r*_g_ ± SE) between the broad depression phenotype and self-reported MDD in the 23andMe data, calculated using the LD Score regression method, was 0.79 ± 0.07 and highly significant (*p* = 5.70 × 10^–28^). In the bivariate meta-analyses of the broad depression phenotype and self-reported MDD, three loci (*NEGR1*, *MAT2B, MUC21*) reached genome-wide significance and all were replicated. *MUC21* is novel for depression (Fig. 1a, Table 1, Supplementary Table 1, Supplementary Figure 1).

**Table 1:** Loci resulting from bivariate discovery meta-analyses of the broad depression phenotype with self-reported MDD, recurrent MDD, bipolar disorder and schizophrenia and replication for broad depression in the UK Biobank (UKB)

gSNP	A1	A2	EAF (UKB)	Chr	Position Ch37/hg19	Nearest gene^a^	*P* value Broad depression^b^ (*N* = 70,017)	*P* value of 4 bivariate GWASs	*P* value bivariate discovery	*P* value (1-sided) UKB replication (*N* = 322,580)	*P* value combined (discovery+replication)	ED^c^	Novel for^d^
sMDD (*N* = 307,354)													
rs2422320	T	C	0.43	1	73293332	*NEGR1*	4.32×10^–4^	2.76×10^–6^	5.25×10^–9^	**3.66×10**^**–5**^	1.52×10^–11^	---	^h[5, 9]^
rs7714851	T	C	0.52	5	164475774	*MAT2B*	4.24×10^–6^	1.09×10^–6^	1.60×10^–11^	**5.35×10**^**–7**^	8.03×10^–16^	---	^h[5, 9]^
rs9368649	A	G	0.14	6	30938883	*MUC21*	3.18×10^–5^	1.14×10^–5^	1.36×10^–8^	**4.75×10**^**–3** e^	9.97×10^–9^	---	Depression
rMDD (*N* = 10,640)													
rs9323497	T	C	0.05	14	67873128	*PLEK2*	3.27×10^–8^	2.21×10^–4^	3.05×10^–10^	4.12×10^–1^	4.48×10^–9^	+++	
BPD (*N* = 16,731)													
rs16836940^g^	A	G	0.22	1	150416913	*RPRD2*	4.63×10^–6^	5.32×10^–4^	1.28×10^–8^	2.96×10^–1^	6.42×10^–8^	+++	BPD
rs2535629	A	G	0.33	3	52833219	*ITIH3*	1.89×10^–4^	8.20×10^–7^	4.94×10^–9^	**6.60×10**^**–3**^	1.10×10^–9^	---	^h[15]^
rs4765914^f^	T	C	0.79	12	2420377	*CACNA1C*	1.25×10^–4^	6.78×10^–6^	3.77×10^–9^	4.24×10^–2^	5.25×10^–9^	+++	^h[15]^
SCZ (*N* = 150,064)													
rs12407717	T	C	0.13	1	30426014	*MATN1*	7.49×10^–6^	1.45×10^–4^	4.91×10^–9^	1.82×10^–1^	1.89×10^–8^	++-	SCZ
rs12128108	T	C	0.23	1	50293421	*AGBL4*	3.35×10^–5^	9.18×10^–6^	1.32×10^–9^	6.65×10^–2^	2.62×10^–9^	+++	SCZ
rs2318763^g^	A	G	0.17	1	150115974	*VPS45*	1.91×10^–4^	5.84×10^–9^	1.48×10^–11^	1.69×10^–1^	4.84×10^–11^	+++	
rs7597593	T	C	0.62	2	185533580	*ZNF804A*	1.89×10^–4^	1.47×10^–9^	4.80×10^–12^	**2.14×10**^**–2**^	2.53×10^–12^	+++	Depression
rs2535629	A	G	0.33	3	52833219	*ITIH3*	1.89×10^–4^	1.33×10^–8^	8.95×10^–11^	**6.60×10**^**–3**^	2.04×10^–11^	---	^h[15]^
rs1966136	A	C	0.41	3	61153578	*FHIT*	1.03×10^-7^	8.00×10^-4^	2.52×10^–9^	**6.45×10**^**–4**^	6.13×10^–11^	---	^h[6]^, SCZ
rs911186	A	G	0.23	6	27150599	*MIR3143*	4.21×10^–4^	1.93×10^–14^	3.90×10^–16^	**2.00×10**^**–3**^	3.50×10^–18^	+++	Depression
rs1265099	A	G	0.41	6	31105413	*PSORS1C2*	1.25×10^–4^	1.62×10^–6^	1.06×10^–9^	**6.90×10**^**–3**^	2.39×10^–10^	+++	Depression, SCZ
rs389883	T	G	0.68	6	31947460	*STK19*	9.65×10^–4^	1.35×10^–7^	1.38×10^–9^	**2.37×10**^**–5**^	1.33×10^–12^	+++	Depression, SCZ
rs4976976	A	G	0.40	8	143311653	*TSNARE1*	5.14×10^–4^	1.19×10^–10^	2.28×10^–12^	9.25×10^–2^	4.36×10^–12^	---	
rs2026194	T	C	0.24	9	85082899	*SPATA31D1*	4.00×10^–4^	5.40×10^–6^	1.09×10^–8^	**3.62×10**^**–3**^	1.74×10^–9^	+++	Depression, SCZ
rs10774037^f^	A	G	0.79	12	2420526	*CACNA1C*	6.62×10^–5^	2.47×10^–9^	6.40×10^–12^	4.48×10^–2^	7.94×10^–12^	---	^h[15]^
rs2182139	T	C	0.75	14	60149233	*RTN1*	2.38×10^–4^	9.19×10^–6^	9.97×10^–9^	**1.86×10**^**–3**^	8.31×10^–10^	+++	Depression, SCZ
rs4906335	A	C	0.28	14	104021141	*BAG5*	5.78×10^–4^	1.82×10^–12^	4.52×10^–14^	**7.25×10**^**–4**^	9.42×10^–16^	+++	^h[9]^
rs4843613	T	C	0.83	16	87489698	*ZCCHC14*	4.13×10^–5^	2.00×10^–5^	3.40×10^–9^	4.48×10^–2^	4.70×10^–9^	+++	SCZ
rs9951150	A	G	0.46	18	52821124	*TCF4*	5.73×10^–4^	2.31×10^–6^	3.70×10^–8^	**4.64×10**^**–3**^	7.08×10^–9^	---	Depression, SCZ

*gSNP* genome-wide significant index SNP based on bivariate discovery, *A1* effect allele, *A2* other allele, *EAF* effect allele frequency, *ED* effect direction, *MDD* major depressive disorder, *BPD* bipolar disorder, *SCZ* schizophrenia, *sMDD* self-reported MDD, *rMDD* recurrent MDD

^a^Nearest genes were based on refseq genes (build 37). Note that reporting the nearest gene as the name of the locus is a convention and by no means implies causality. See LocusZoom plots in Supplementary Figure 1 for other genes located within the loci, and the post-GWAS analyses for prioritization of potentially causal genes

^b^Combination of depressive symptoms meta-GWAS from the CHARGE consortium and MDD meta-GWAS from the PGC consortium

^c^ED: Effect direction shows the effect directions of the SNP for: 1) the broad depression phenotype; 2) (i) self-reported MDD, (ii) recurrent MDD, (iii) bipolar disorder or (iv) schizophrenia, and; 3) broad depression in the UK Biobank, for the effect alleles (A1)

^d^For BPD and SCZ this is based on the genome-wide significant bivariate discovery *p* value; for Depression this is based on the significant directional consistent replication and genome-wide significant *p* value of discovery and replication combined

^e^rs9368649 was not available in UKB, instead rs2894052 (*r*^2^ = 1) was used as a proxy

^f^rs4765914 and rs10774037 are very close to each other and in very high LD (*r*^2^ = 0.99)

^g^rs16836940 and rs2318763 are in moderate LD (*r*^2^ = 0.39)

^h^Previously reported by others and replicated in our study.

Only *p*-values of replicated SNPs are in bold, that is: (1) 1-sided *p*  < 0.05; (2) the effect was directionally consistent, and (3) the combined discovery and replication meta-analysis *p* value was more significant than its corresponding bivariate GWAS

### Broad depression phenotype with recurrent MDD

The genetic correlation between the broad depression phenotype and recurrent MDD was 0.24 ± 0.08 (*p* = 1.71 × 10^–59^). In the bivariate meta-GWAS of broad depression phenotype and recurrent MDD, the *PLEK2* locus reached genome-wide significance (*p* = 3.05 × 10^–10^) but was not replicated (Fig. 1b, Table 1, Supplementary Table 1).

### Broad depression phenotype with bipolar disorder

The genetic correlation between the broad depression phenotype and bipolar disorder was 0.53 ± 0.09 (*p* = 5.14 × 10^–10^). The bivariate meta-GWAS analysis identified three loci (*RPRD2*, *ITIH3, CACNA1C*) of which *ITIH3* replicated (Fig. 1c, Table 1, Supplementary Table 1, Supplementary Figure 1). The *ITIH3* locus was previously reported [15].

### Broad depression phenotype with schizophrenia

The genetic correlation of the broad depression phenotype with schizophrenia was 0.57 ± 0.05 (*p* = 1.61 × 10^–28^). In the bivariate meta-GWAS analysis with schizophrenia, 16 loci reached bivariate genome-wide significance of which 10 replicated (*ZNF804A, ITIH3, FHIT, MIR3143, PSORS1C2, STK19, SPATA31D1, RTN1, BAG5, TCF4*) and 6 did not (*MATN1, AGBL4, VPS45, TSNARE1, CACNA1C, ZCCHC14*) (Fig. 1d, Table 1, Supplementary Table 1, Supplementary Figure 1). Three replicated loci were previously reported (*ITIH3* [15], *FHIT* [6], *BAG5* [9]), but the other seven (*ZNF804A, MIR3143, PSORS1C2, STK19, SPATA31D1, RTN1, TCF4*) were novel for depression. Although the *TCF4* locus was previously reported by Wray et al. [9], our top SNP is an independent signal (*r*^2^ = 0.004) at this same locus (Supplementary Figure 1). For the *ITIH3* locus, the same SNP was found as for the bivariate GWAS with bipolar disorder (Fig. 1c, d, Table 1).

### Bivariate negative control GWAS analyses

Bivariate LD score regression confirmed that none of the six control outcomes showed a significant genetic correlation. In bivariate GWASs we observed a maximum of only 2 genome-wide significant loci with broad depression: 0 for femoral neck and lumbar spine bone mineral density, 1 for age-related macular degeneration, and 2 for forearm bone mineral density, breast and prostate cancer (Supplementary Table 2). Thus, from six control phenotypes, we identify seven genome-wide significant findings, indicating a false positive rate of approximately 1 locus per bivariate analysis.

### Post-GWAS analysis

#### In silico sequencing analysis

Of the total of 13 replicated loci, 2 genome-wide significant index SNPs (gSNPs) were in LD (*r*^2^ > 0.50) with 10 nonsynonymous SNPs (nsSNP), 9 of which were in LD with rs2535629 (*ITIH3*) and located in 7 different genes, 3 of which belonged to the inter-alpha-trypsin inhibitor heavy chain gene family (*ITIH1*, *ITIH3*, *ITIH4*). Two nsSNPs, rs1029871 mapping to *NEK4* and rs678 to *ITIH1*, were considered ”deleterious” by SIFT and “possibly”, respectively, ”probably damaging” by PolyPhen. The nsSNP rs437179 located within the *SKIV2L* gene was in high LD with the other gSNP rs389883 (*STK19*) (Table 2).

**Table 2:** Nonsynonymous SNPs in LD (*r*^2^ ≥ 0.50) with the replicated gSNPs

gSNP	Nonsynonymous SNP linked with gSNPs	CHR	BP	A1	A2	LD (*r*^2^)	Gene
rs2535629	rs4434138	3	52556890	A	G	0.56	*STAB1*
	rs11177	3	52721305	G	A	0.75	*GNL3*
	rs2289247	3	52727257	G	A	0.73	*GNL3*
	rs6617	3	52740182	C	G	0.73	*SPCS1*
	rs1029871^a, b^	3	52797634	G	C	0.75	*NEK4*
	rs678^a, c^	3	52820981	A	T	0.84	*ITIH1*
	rs1042779	3	52821011	A	G	0.84	*ITIH1*
	rs3617	3	52833805	C	A	0.64	*ITIH3*
	rs4687657	3	52852538	G	T	0.55	*ITIH4*
rs389883	rs437179	6	31929014	A	C	0.99	*SKIV2L*

^a^Deleterious (SIFT)

^b^Possibly damaging (PolyPhen)

^c^Probably damaging (PolyPhen)

Combining our 13 replicated gSNPs with those of established depression loci from the literature yielded a total of 56 gSNPs (Supplementary Material). Together with the 2 gSNPs containing nsSNPs in 8 genes described above, these 56 gSNPs included an additional 4 gSNPs in LD (*r*^2^ > 0.50) with 4 nsSNPs mapping to 4 genes, i.e., a total of 12 genes containing nsSNPs. Our analyses also revealed 1962 SNPs that are in high LD (*r*^2^ > 0.8) with the 56 gSNPs which map to 70 genes (Supplementary Table 3). The 12 genes containing nsSNPs in combination with the latter 70 genes in high LD, genes closest to the 56 gSNPs and their expression quantitative trait locus (eQTL) genes (Supplementary Table 4) were used in the functional network and enrichment analysis described below.

#### In silico lookup of GWAS associations

This analysis returned a number of psychiatric and somatic traits and diseases (Supplementary Table 5).

#### eQTL analysis

Table 3 shows the findings from the *cis*-eQTL analysis of our 13 replicated gSNPs. We observed a total of 34 genes that were *cis*-eQTLs in the three databases, 11 in BRAINEAC, 14 in GTEx and 16 in the Westra et al. [32] peripheral blood database. Six of the 34 genes (*ITIH4*, *GNL3*, *PRSS16, SKIV2L, XRCC3* and *KLC1*) had eQTLs in at least two of the three databases. Of these genes, *ITIH4* and *GNL3* also contain nsSNPs in LD with rs2535629 (Table 2). Interestingly, *SKIV2L* is an eQTL for rs389883, which was also in high LD with a nsSNP in this gene (Table 2).

**Table 3:** eQTL analysis results for the 13 replicated gSNPs

SNP	CHR	BP	Allele	eQTL lookup in three databases		
				Westra et al. [32], FDR < 0.05	BRAINEAC, *p* < 1 × 10^–5^	GTEx, FDR < 0.05
rs2535629	3	52833219	G/A	*SPCS1*, ***ITIH4***	***ITIH4***, ***GNL3****, GLT8D1, SNORD19B*	***ITIH4***, *PPM1M*, ***GNL3***
rs911186	6	27150599	A/G	*HIST1H2BK,* ***PRSS16***	*ZNF389, BTN2A1,BTN2A2*	***PRSS16****, ZNF204P*
rs9368649	6	30938883	A/G	*VARSL*		
rs1265099	6	31105413	G/A	*HCG27*	*PSORS1C2*	*C4A*
rs389883	6	31947460	G/T	*DOM3Z, HSPA1B,* ***SKIV2L***	*NFKBIL1*	*CYP21A1P, HLA-C, HLA-DRB5, WASF5P, XXbac-BPG248L24.12,* ***SKIV2L****, HLA-DRB1*
rs2182139	14	60149233	C/T	*RTN1, C14orf100*		
rs4906335	14	104021141	C/A	*BAG5,* ***XRCC3****,****KLC1****, EIF5, CKB*	***KLC1****,C14orf153*	***XRCC3***

Genes with eQTLs in multiple databases are in bold

#### Functional network and enrichment analysis

Functional network and enrichment analysis that included all four prioritized gene sets (see Supplementary Materials for details) showed enrichment of Gene Ontology (GO) terms involved in the Ras superfamily, the Rab family and GTPase activity, as well as terms involved in the epigenetic machinery and chromatin function (Supplementary Table 6). If limited to the closest genes, the enrichment for Ras and Rab protein functions became even stronger. In addition, terms related to nervous system development and neuronal differentiation became significant (Supplementary Table 7). Limiting the input query genes to those with strong functional evidence based on coding SNPs and/or eQTLs confirmed the involvement of epigenetic regulation and chromatin function (Supplementary Tables 8 and 9). Based on the results of the four bivariate GWASs, DEPICT identified one gene, four nervous system-related tissues and one gene-set that were significantly enriched (false discovery rate (FDR) < 0.05). The bivariate GWAS with schizophrenia yielded enrichment of three tissues: hypothalamus middle, hypothalamo-hypophyseal system and neurosecretory systems (Supplementary Figure 2). The one with self-reported MDD showed enrichment in basal ganglia (Supplementary Figure 3). The identified gene (*SMARCA2*) and gene-set (SWI/SNF-type complex) provide converging evidence in line with the involvement of epigenetic regulation and chromatin function because the encoded protein is part of the large ATP-dependent SWI/SNF chromatin remodeling complex.

## Discussion

This study combined a GWAS of the broad depression phenotype with the GWAS results of self-reported MDD, recurrent MDD, bipolar disorder and schizophrenia and found substantial genetic correlations with self-reported MDD (0.79), bipolar disorder (0.53) and schizophrenia (0.57), and a more moderate but still highly significant trans-ethnic genetic correlation with recurrent depression (0.24). We identified 20 independent loci in the 4 subsequent bivariate GWASs, of which 13 replicated in the UK Biobank. Eight of these were novel for depression. In the bivariate GWAS with self-reported MDD, we reported one novel genetic locus mapping to *MUC21*. The bivariate GWAS with schizophrenia yielded 16 loci (listed in Table 1), 10 of which replicated, including 7 novel for depression: *ZNF804A, MIR3143, PSORS1C2, STK19, SPATA31D1, RTN1* and *TCF4*. Out of the five previously reported loci, *NEGR1* and *MAT2B* were previously reported as associated with self-reported MDD [5], *FHIT* with broad depression [6], *ITIH3* was detected in a cross-disorder GWAS [15] and finally *BAG5* was identified in the recent PGC meta-GWAS for MDD [9].

In order to prioritize particular genes within the 13 identified loci for their likely causal involvement, we performed a series of post-GWAS analyses investigating whether our lead SNPs were in high LD with coding SNPs, were associated with gene expression in blood or brain or had previously been reported in the literature to be associated with similar or other complex traits. These analyses yielded a number of candidates with strong evidence for their functional involvement in the nervous system and brain development. Thus, they may be involved in the pathogenesis of psychiatric disorders including depression (see Supplementary Materials).

One example is the inter-alpha-trypsin inhibitor heavy chain gene family (*ITIH1*, *ITIH3*, *ITIH4*), which has been associated with major psychiatric disorders including MDD [15], bipolar disorder [15, 33] and schizophrenia [15, 33]. We confirmed their likely functional involvement based on LD with nsSNPs for *ITIH1*, *ITIH3* and *ITIH4*, whereas *ITIH4* was an eQTL in both blood and brain across all three databases. Furthermore, LD with a nsSNP pointed to *SKIV2L* as a likely causal candidate for depression, additionally supported by eQTL evidence in blood and brain.

For the functional network and enrichment analyses, we included all our prioritized genes based on our replicated SNPs in combination with those of established depression loci from the literature. The findings showed enrichment of GO terms involved in the Ras superfamily, the Rab family and GTPase activity. The Rab protein family is involved in clathrin-mediated endocytosis, a mechanism governing cellular membrane and protein trafficking, which has been implicated in the pathophysiology of psychotic disorders through synaptic dysfunction, white matter changes and aberrant neurodevelopment [34]. Thus, the current evidence also implicates depression, in line with earlier evidence that activators of the Ras superfamily of GTPases, Raf1 and B-Raf, are dysregulated in postmortem brains of suicide cases [35]. Other enriched GO terms highlighted the epigenetic machinery and chromatin function as well as nervous system development and neuronal differentiation. These latter enrichments were confirmed by our DEPICT analyses and by post-GWAS results from the most recent PGC MDD meta-GWAS [9].

An important asset of our study is that we used most of the currently available data and exploited the biological pleiotropy among psychiatric disorders [36] to identify eight novel loci for broad depression replicated in a large population-based cohort. On the other hand, one potential limitation of our four bivariate analyses is that they do not allow identification of loci that are specific for depression. We showed that our strategy of combined analysis of the broad depression phenotype with correlated psychiatric disorders or traits was most successful for the largest sample sizes (those for self-reported MDD and schizophrenia). In addition, although not the primary aim of our study, we also identified one novel locus for bipolar disorder and nine for schizophrenia (Table 1), although these need to be replicated. One limitation of our approach of using GWAS summary statistics in our analyses is that we could not explore potential heterogeneity of our results by stratifying, for example, for age and sex. However, it would be interesting for future studies to explore whether effects of established loci, including those we have now replicated using the UK Biobank results, may vary according to age, sex or even depressive subtype. Another point of discussion may be our strategy to avoid the identification of false positive loci. We took the following precautions: in the bivariate GWAS discovery phase we applied a strict significance criterion of *p* < 5 × 10^–8^ and demanded a nominal and directionally consistent effect in both contributing GWAS samples (*p* < 0.001). In the replication phase, a SNP was only considered replicated if a one-sided test was nominally significant (*p*_one sided_ < 0.05), i.e., the effect was directionally consistent, and the combined meta-analysis of the discovery and replication yielded a *p* value more significant than the corresponding bivariate *p* value. Although these are sound precautions similar to those applied in previous recent work [37], it can still be argued that the replication phase may have yielded 1–2 loci merely based on chance. Thus, we applied, post-hoc, an additional FDR analysis to our replication *p* values. Reassuringly, all 13 replicated SNPs showed an FDR < 0.05 confirming the appropriateness and strictness of our replication criteria.

The substantial genetic correlations within depression (ranging from the mild depression continuum to more severe recurrent MDD) and across psychiatric disorders (i.e., bipolar disorder, schizophrenia) suggest that psychiatric illnesses may differ, in part, quantitatively rather than qualitatively from each other, and cross-disorder (dimensional) [24] analysis approaches may be the best way forward. It has even been argued that genetic variants that are specific to a single diagnostic category are unlikely to exist and that our reliance on separate diagnoses actually hampers progress [36]. As evidenced by the results of our study, cross-disorder analyses yield novel genetic loci and will extend our understanding of overlapping mechanisms among the different psychiatric disorders.

## Web resources

Recurrent MDD, bipolar disorder and schizophrenia summary data: http://www.med.unc.edu/pgc/downloads. UK Biobank depression summary data: https://datashare.is.ed.ac.uk/handle/10283/3036. Blood eQTL browser: http://genenetwork.nl/bloodeqtlbrowser. UK Brain Expression Consortium (UKBEC): http://www.braineac.org/. GTEx: http://www.gtexportal.org/home/. Ensembl genome browser: http://www.ensembl.org. DEPICT: www.broadinstitute.org/depict. Online methods: eLX package: https://sites.google.com/site/multivariateyihsianghsu/. LDSC software: https://github.com/bulik/ldsc.

## Supplementary information

Supplementary figures

Supplementary methods and Materials

Supplementary Table 1

Supplementary Table 2

Supplementary Table 3

Supplementary Table 4

Supplementary Table 5

Supplementary Table 6

Supplementary Table 7

Supplementary Table 8

Supplementary Table 9
