# Supplementary material for: Bivariate genome-wide association analyses of the broad depression phenotype combined with major depressive disorder, bipolar disorder or schizophrenia reveal eight novel genetic loci for depression
Source: Mol Psychiatry. 2019 Jan 9;25(7):1420–9. doi: 10.1038/s41380-018-0336-6 (PMC7303007; doi:10.1038/s41380-018-0336-6)
Supplement: Supplementary file 1 — Supplementary figures [file 41380_2018_336_MOESM1_ESM.pdf]

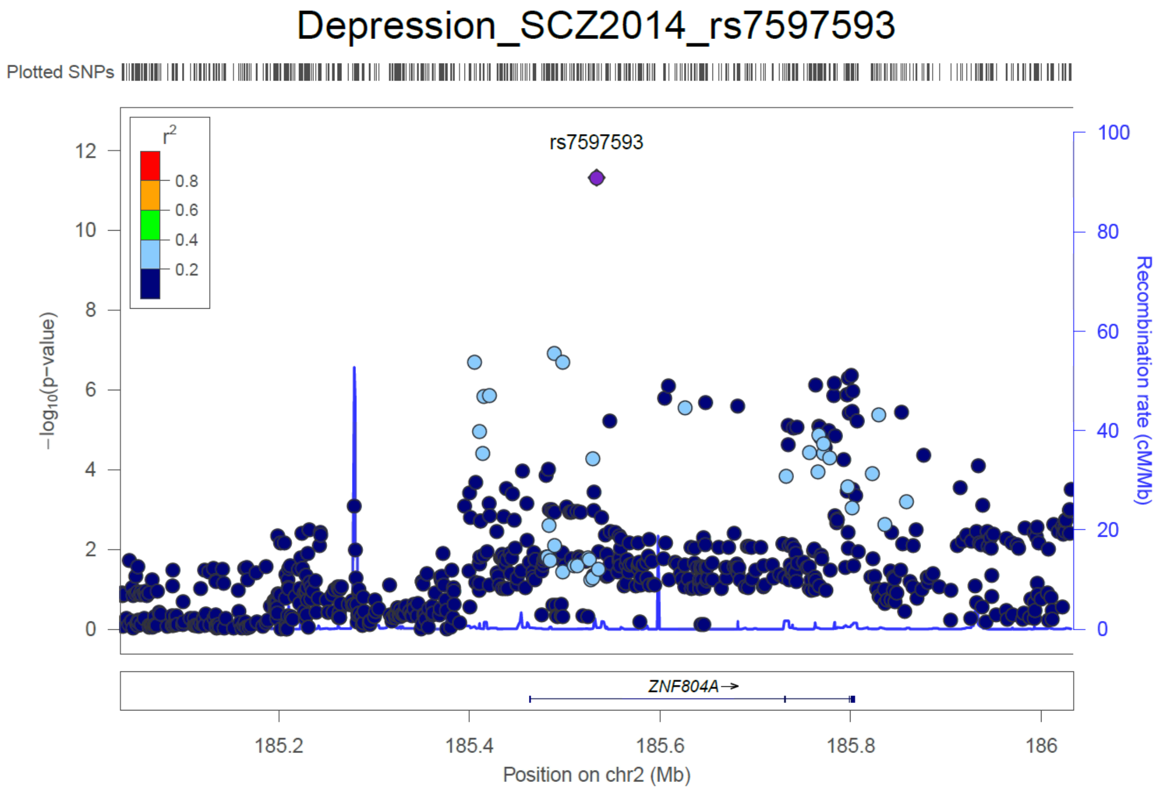
Depression_SCZ2014_rs7597593
Plotted SNPs
rs7597593
r2
0.8
0.6
0.4
0.2
−log10(p−value)
Recombination rate (cM/Mb)
ZNF804A→
185.2
185.4
185.6
185.8
186
Position on chr2 (Mb)

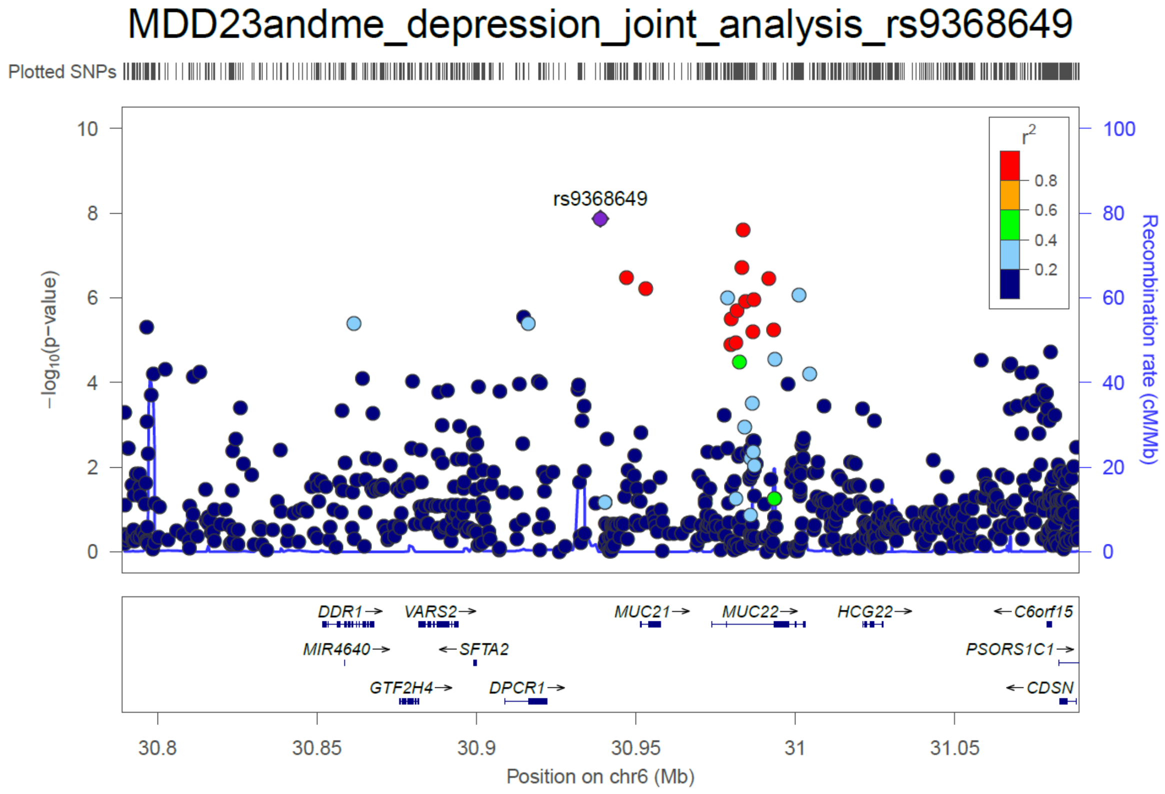
MDD23andme_depression_joint_analysis_rs9368649
Plotted SNPs
rs9368649
r2
0.8
0.6
0.4
0.2
−log10(p−value)
Recombination rate (cM/Mb)
DDR1→
VARS2→
MUC21→
MUC22→
HCG22→
←C6orf15
MIR4640→
←SFTA2
PSORS1C1→
GTF2H4→
DPCR1→
←CDSN
30.8
30.85
30.9
30.95
31
31.05
Position on chr6 (Mb)

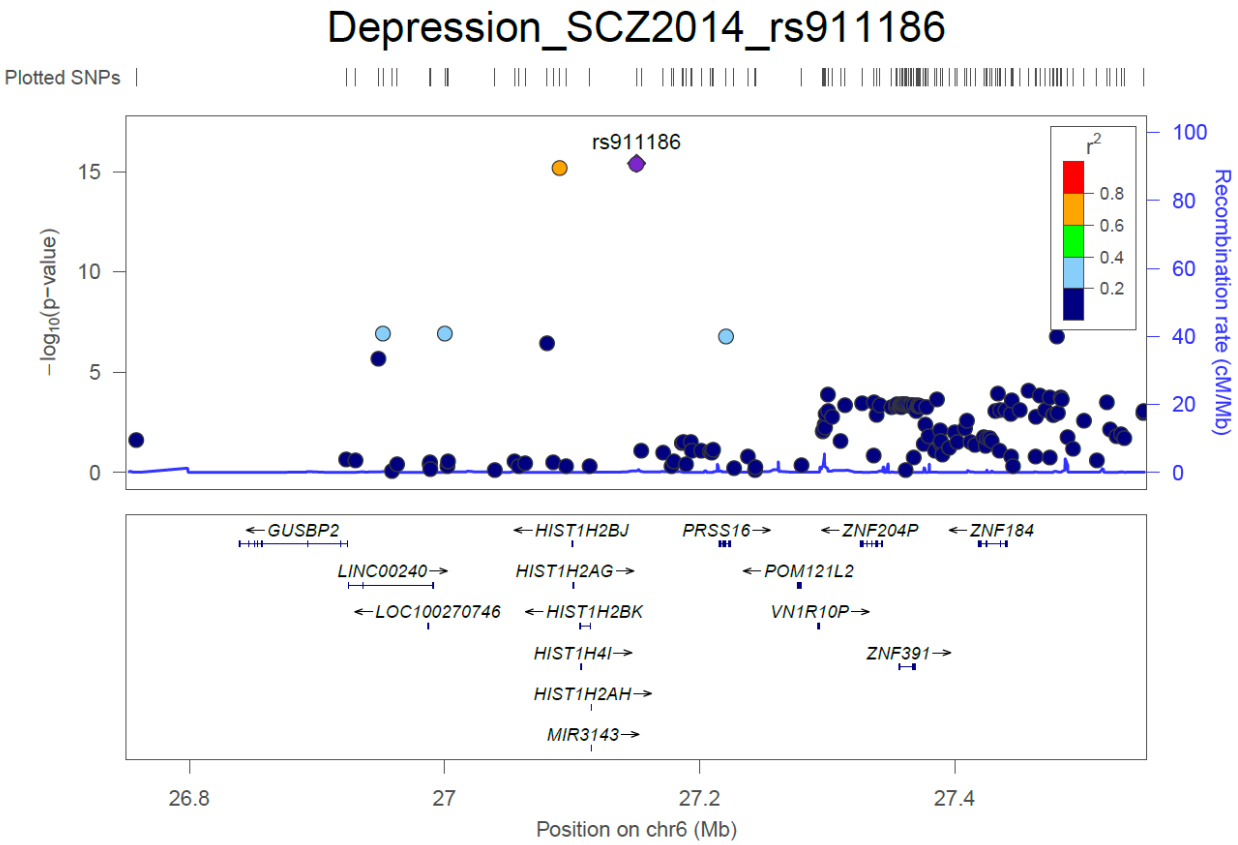
Depression_SCZ2014_rs911186
Plotted SNPs
rs911186
−log10(p−value)
Recombination rate (cM/Mb)
r2
0.8
0.6
0.4
0.2
GUSBP2
LINC00240
LOC100270746
HIST1H2BJ
HIST1H2AG
HIST1H2BK
HIST1H4I
HIST1H2AH
MIR3143
PRSS16
POM121L2
VN1R10P
ZNF204P
ZNF184
ZNF391
26.8
27
27.2
27.4
Position on chr6 (Mb)

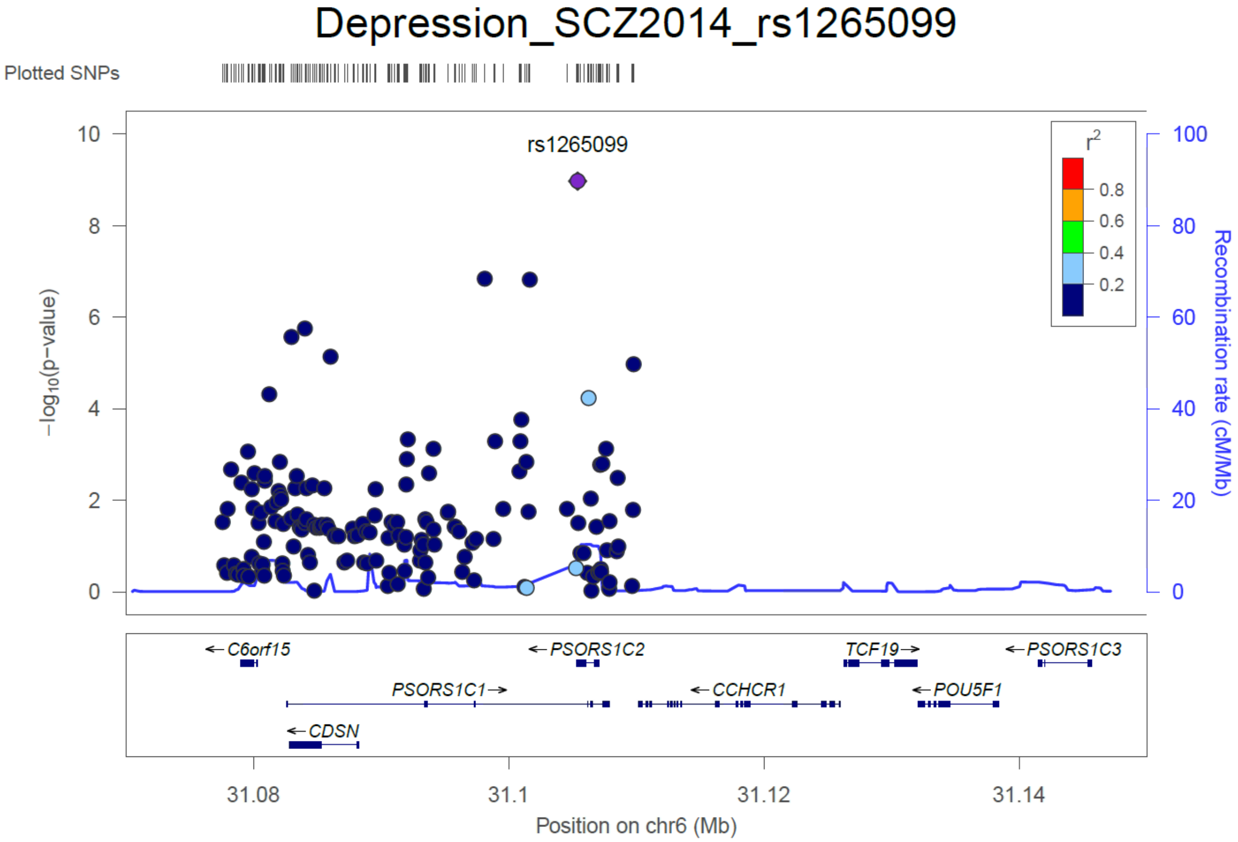
Depression_SCZ2014_rs1265099
Plotted SNPs
rs1265099
−log10(p−value)
Recombination rate (cM/Mb)
r2
0.8
0.6
0.4
0.2
C6orf15
PSORS1C1
CDSN
PSORS1C2
CCHCR1
TCF19
POU5F1
PSORS1C3
31.08
31.1
31.12
31.14
Position on chr6 (Mb)

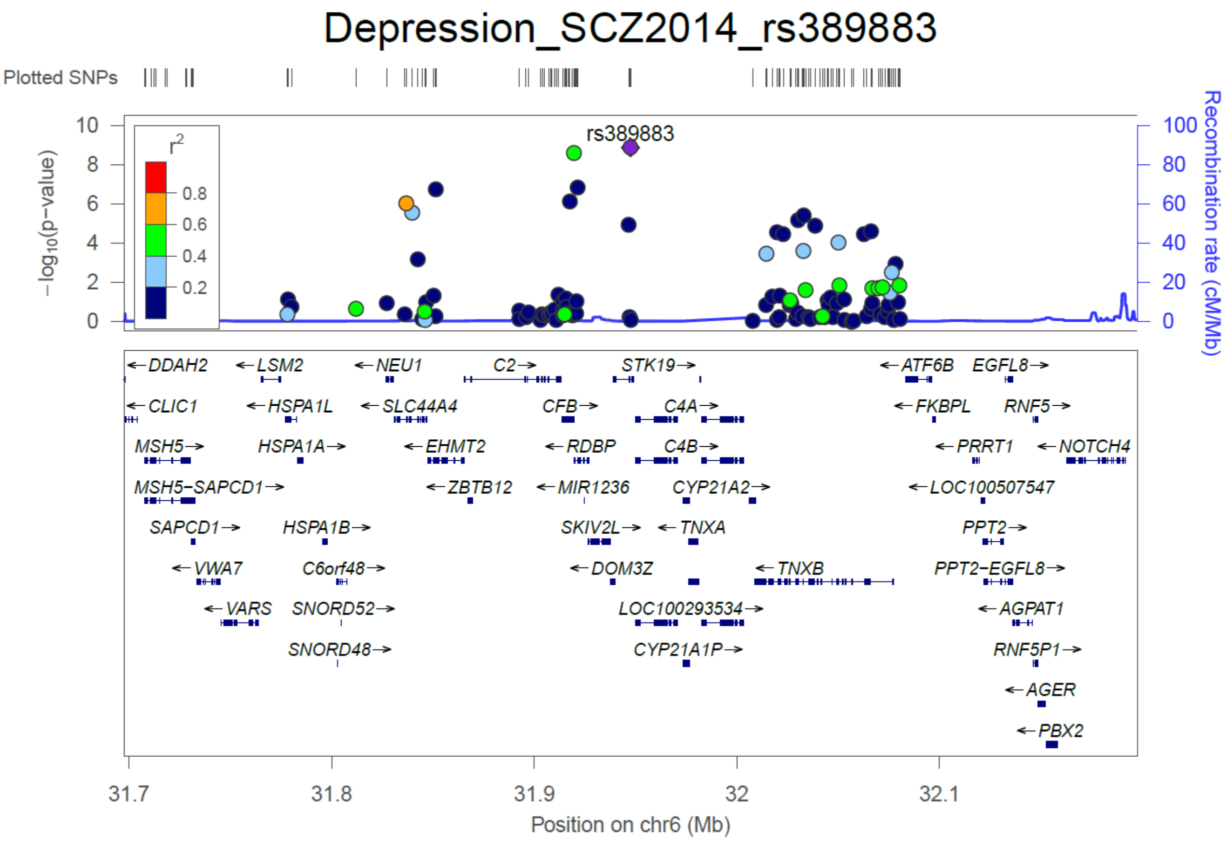
Depression_SCZ2014_rs389883
Plotted SNPs
rs389883
r2
0.8
0.6
0.4
0.2
−log10(p−value)
Recombination rate (cM/Mb)
DDAH2
LSM2
NEU1
C2
STK19
ATF6B
EGFL8
CLIC1
HSPA1L
SLC44A4
CFB
C4A
FKBPL
RNF5
MSH5
HSPA1A
EHMT2
RDBP
C4B
PRRT1
NOTCH4
MSH5−SAPCD1
ZBTB12
MIR1236
CYP21A2
LOC100507547
SAPCD1
HSPA1B
SKIV2L
TNXA
PPT2
VWA7
C6orf48
DOM3Z
TNXB
PPT2−EGFL8
VARS
SNORD52
LOC100293534
AGPAT1
SNORD48
CYP21A1P
RNF5P1
AGER
PBX2
31.7
31.8
31.9
32
32.1
Position on chr6 (Mb)

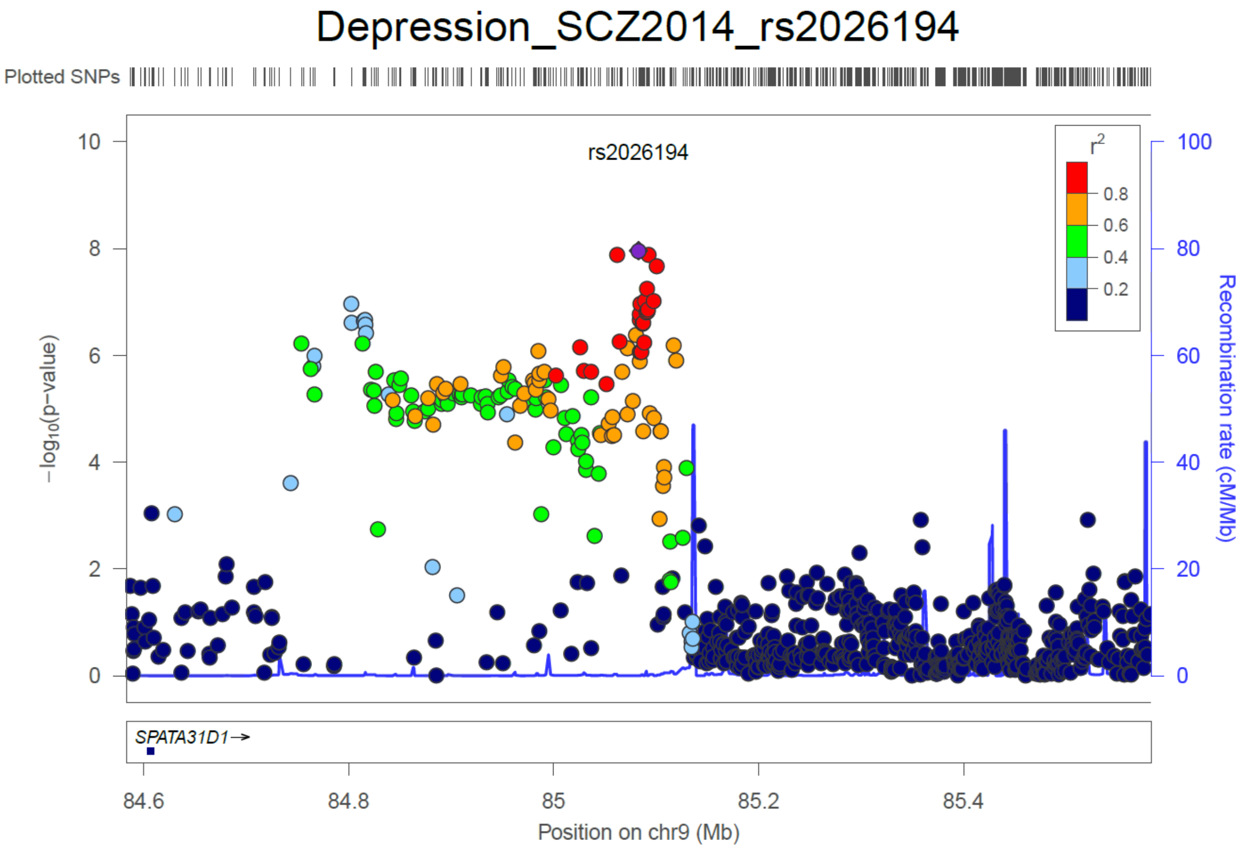
Depression_SCZ2014_rs2026194
Plotted SNPs
rs2026194
r2
0.8
0.6
0.4
0.2
−log10(p−value)
Recombination rate (cM/Mb)
SPATA31D1
84.6
84.8
85
85.2
85.4
Position on chr9 (Mb)

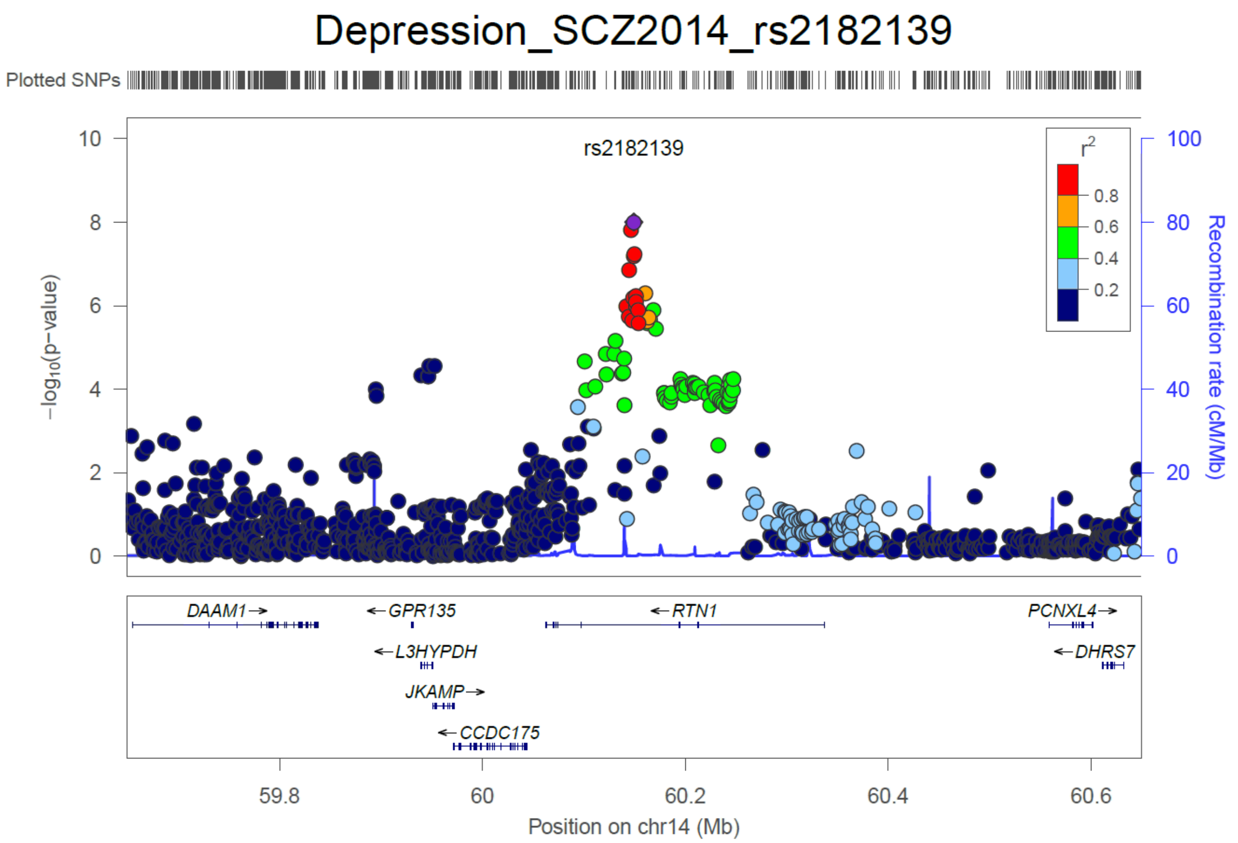
Depression_SCZ2014_rs2182139
Plotted SNPs
rs2182139
r2
0.8
0.6
0.4
0.2
−log10(p−value)
Recombination rate (cM/Mb)
DAAM1
GPR135
RTN1
PCNXL4
L3HYPDH
DHRS7
JKAMP
CCDC175
59.8
60
60.2
60.4
60.6
Position on chr14 (Mb)

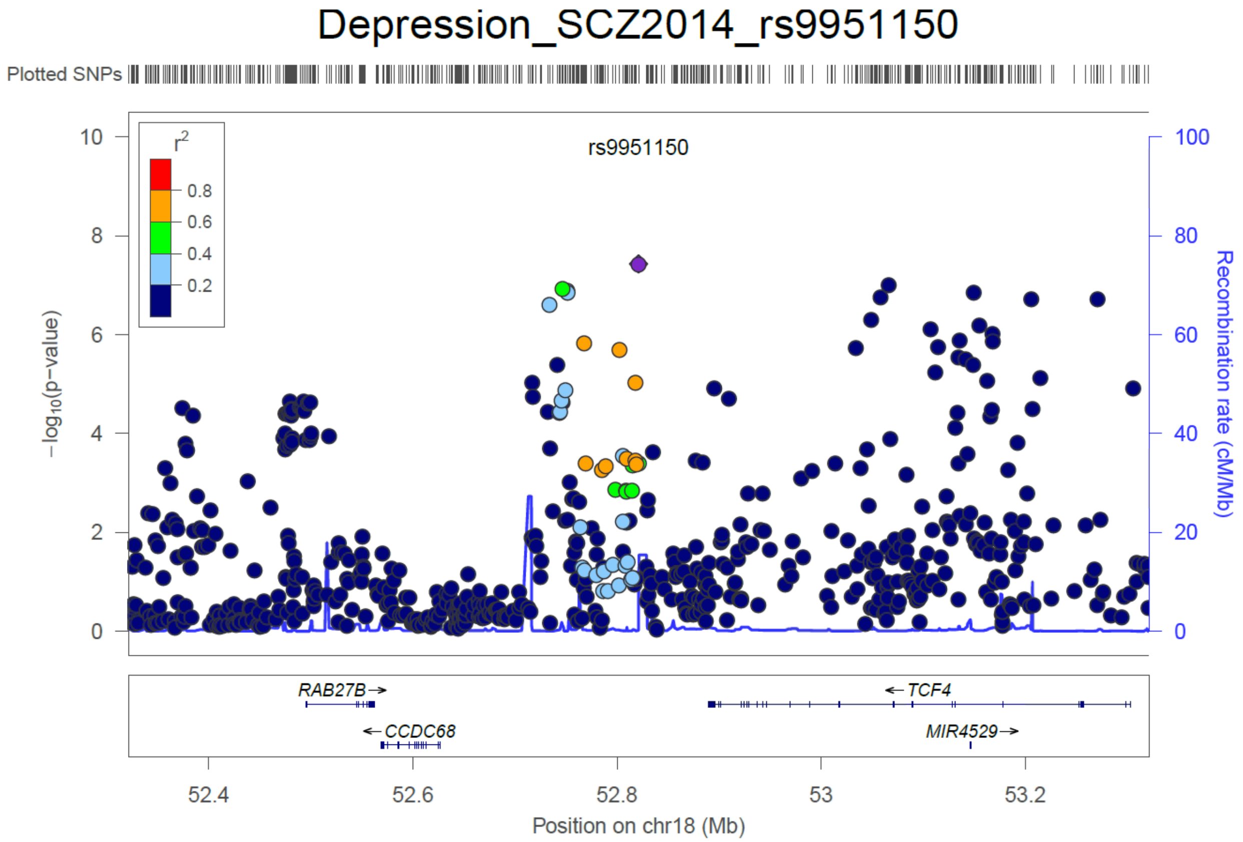
Depression_SCZ2014_rs9951150
Plotted SNPs
rs9951150
r2
0.8
0.6
0.4
0.2
−log10(p−value)
Recombination rate (cM/Mb)
RAB27B
TCF4
CCDC68
MIR4529
52.4
52.6
52.8
53
53.2
Position on chr18 (Mb)

Supplementary Figure 1: Regional association plots of the eight genetic loci novel for depression from the 13 replicated gSNPs

NOTE: The lead SNP and the psychiatric traits are indicated in the top line. In the plots rs-numbers of the lead SNPs are given. Color coding reflects the LD (r^2) of the SNP with this lead SNP according to the legend. The left y-axis represents -log10(p-value) of association; the right y-axis indicates recombination rate. The x-axis depicts the position on the chromosome (Mb) and lists known genes near the locus.

Abbreviations: SCZ2014: Schizophrenia 2014 GWAS study, MDD23andme: self-reported MDD from 23andme GWAS study.

a)

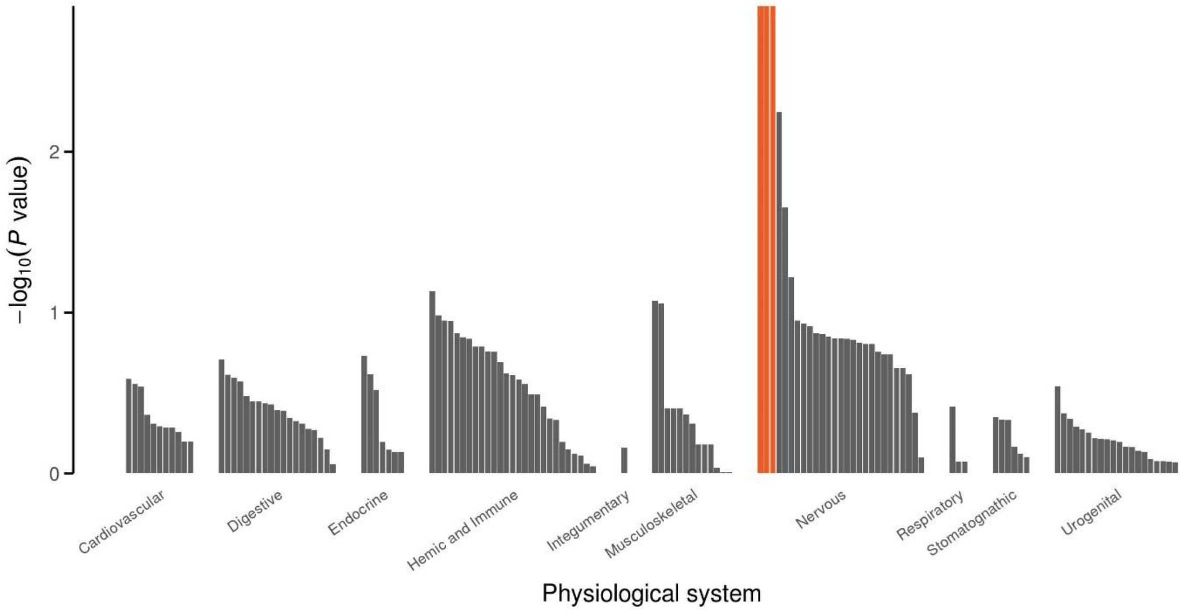

b)

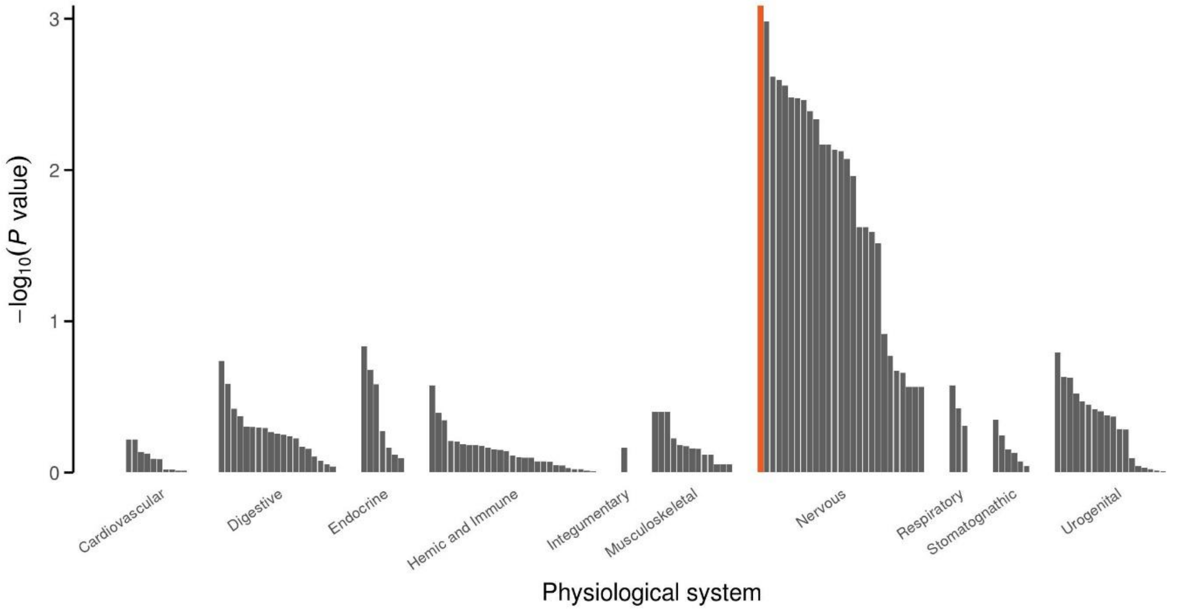

Supplementary Figure 2: DEPICT results of tissue/cell type enrichment analysis using bivariate GWAS analyses of the broad depression phenotype with **a)** schizophrenia, and; **b)** self-reported MDD. The red bars are significant (FDR<0.05).
