# Supplementary material for: Bivariate genome-wide association analyses of the broad depression phenotype combined with major depressive disorder, bipolar disorder or schizophrenia reveal eight novel genetic loci for depression
Source: Mol Psychiatry. 2019 Jan 9;25(7):1420–9. doi: 10.1038/s41380-018-0336-6 (PMC7303007; doi:10.1038/s41380-018-0336-6)
Supplement: Supplementary file 2 — Supplementary methods and Materials [file 41380_2018_336_MOESM2_ESM.pdf]

Supplementary Methods and Materials

Overview of the univariate genome-wide association studies

Broad depression phenotype**:** The meta-GWAS for the broad depression phenotype in the discovery phase comprised 70,017 subjects. This study was itself a combined analysis of the depressive symptoms meta-GWAS from the CHARGE consortium[1] and the MDD meta-GWAS from the PGC.[2] The meta-analysis of MDD consisted of nine studies of 9240 cases meeting international criteria for lifetime MDD and 9519 healthy control subjects. The CHARGE meta-analysis of depressive symptoms included 22 cohorts and comprised 51,258 persons. All subjects were of European descent. The details of the joint GWAS can be found in the original publication.[3]

Self-reported MDD: This is the first successful GWAS for MDD in a population of European origin. The study utilized online self-reported data through 23andMe, Inc., a personal genetics company (https://www.23andme.com/en-int), involving 75,607 cases reporting clinical diagnosis or treatment of depression and 231,747 controls reporting no such diagnosis or treatment.[4]

Recurrent MDD: Involved a study in Chinese women of 5,303 cases with recurrent MDD and 5,337 controls.[5]

Bipolar disorder**:** A study in individuals of European origin comprised of 7,481 cases with bipolar disorder and 9,250 controls.[6]

Schizophrenia: We used the most recent and largest meta-GWAS of schizophrenia that incorporated 36,989 cases and 113,075 controls who were of European and east Asian origin.[7]

The genome-wide genotyping of these studies was performed using Illumina (Illumina, Inc., San Diego, California) or Affymetrix (Affymetrix, Santa Clara, California) platforms. The genotype data for GWASs for the broad depression phenotype was itself a combination of data from the PGC and CHARGE depressive symptoms. The PGC samples were imputed using the CEU (Central Europe) and TSI (Toscani in Italy) HapMap3 reference panels. The CHARGE depressive symptoms and bipolar disorder data were imputed based on reference haplotypes from the HapMap phase 2 CEU sample.[8] This yielded 918,921 and 2,415,422 SNPs, for broad depression and bipolar disorder, respectively, while the genotype data for self-reported MDD, recurrent MDD, and schizophrenia were imputed using the 1000 Genomes project reference panel yielding 13,535,270, 6,242,619 and 9,444,230 SNPs, respectively.[9] In the analyses of the GWASs, imputation, quality control and adjustment to covariates such as age, sex, and population stratification have been done before the final meta-analyses and the details of the procedures can be found in the original publications.[3-7]

Bivariate negative control GWAS analyses

Using publicly available GWAS summary statistics, we conducted LD Score regression and bivariate negative control GWAS analyses with six GWASs of three classes of common complex traits and diseases presumably unrelated to depression: 1) age-related macular degeneration[10]; 2) osteoporosis measured as bone mineral density (BMD) of (i) femoral neck; (ii) lumbar spine and; (iii) forearm[11] and; 3) cancer of (i) breast and (ii) prostate (UK Biobank)[12].

Post-GWAS analyses

We used the post-GWAS pipeline from Vaez et al.[13] to annotate findings of our 13 replicated GWAS SNPs (gSNPs) in combination with those of established depression loci from the literature: 1 SNP reported by Direk et al. (2017),[3] 17 SNPs (from 15 loci) reported by Hyde et

al. (2016)[4]; 2 replicated SNPs reported by Okbay et al. (2016)[14]; and 44 variants reported by Wray et al. (2017).[15] Four variants from Wray et al. (2017)[15] were indels (not SNPs) and hence, were removed. Thus, after removing two duplicates, we ended up with a single, merged set of 71 SNPs. We then clumped the SNPs (1 MB, r^2=0.10) to remove 15 overlapping loci and arrived at a total of 56 gSNPs. These 56 gSNPs were used for downstream analyses, which included *in silico* sequencing, *in silico* lookup of associations with other phenotypes in the GWAS catalogue [16], expression quantitative trait loci analysis and functional network and enrichment analysis.

***In silico* sequencing:** For the *in silico* sequencing, we used the data of the 1000 Genomes Project phase3 release of variant calls based on the 20130502-sequence freeze and alignments, version v5a (Feb. 20th, 2015), and included only the 503 subjects of European ancestry.[9] The Variant Call Format (VCF)[17] files for regions of 1 Mb at either side of each of the 56 gSNPs (see above) were generated using Tabix.[18] Then, the r^2 between each gSNP and all other bi-allelic SNPs within the corresponding 2 Mb area were calculated as a metric of linkage disequilibrium (LD) using PLINK (v1.9).[19] All SNPs in LD ($r^2 > 0.50$) with any of the gSNPs were then annotated by ANNOVAR software[20] (version July 16, 2017, accessed February 4, 2018). The nonsynonymous SNPs revealed in the annotation phase were then characterized for their damaging impact on the corresponding protein using SIFT[21] and PolyPhen[22] prediction scores. These scores were obtained from Ensembl release 91 (accessed February 17, 2018).

***In silico* pleiotropy analysis:** To identify any other trait or outcome associated with the 56 gSNPs, we used the publicly available data of the National Human Genome Research Institute (NHGRI) GWAS Catalog.[16] We checked for pleiotropic effects of all 56 gSNPs as well as all their linked variants with $r^2 > 0.50$ (revealed in the previous phase of *in silico* sequencing) on other complex traits or diseases identified in previous GWAS studies and

listed in GWAS Catalog using ANNOVAR software[20] (version July 16, 2017, accessed February 4, 2018).

Expression quantitative trait loci analysis: To characterize the effect of the 56 gSNPs on the expression of nearby genes, we searched for *cis* expression quantitative trait loci (*cis*-eQTL) effects using a 1 Mb window size surrounding each lead SNP in three databases representing findings from the largest eQTL-mapping studies in peripheral blood or brain tissues using strict levels of significance. In the Westra *et al.*[23] eQTL database in peripheral blood, we used an FDR<0.05. In the Almanac (Braineac) eQTL database from brain tissue in 10 regions[24] we used a significance level of $p<1\times10^{-5}$ as recommended by the UK Brain Expression Consortium. eQTLs with $p<1\times10^{-5}$ in at least one of the 10 brain regions or all the 10 regions combined were considered significant. From the Genotype-Tissue Expression (GTEx) portal we used data from 13 brain regions with an FDR<0.05.[25] The GTEx dataset release V7 (dbGaP Accession phs000424.v7.p2) was accessed from the GTEx Portal (see web resources).

Functional network and enrichment analysis: As the final step of the post-GWAS pipeline, we combined four prioritized gene sets into a single query gene list. For this analysis, we excluded corresponding gene names of four gSNPs (rs115507122, rs9368649, rs1265099 and rs389883) and all their linked variants that mapped to the major histocompatibility complex (MHC) region on chromosome 6 to prevent bias towards enrichment of immunological pathways due to the extensive LD in this region. The query gene list thus consisted of: (i) closest genes to the 52 gSNPs; (ii) genes mapping to the nonsynonymous SNPs in high LD (r^2>0.50) with the corresponding gSNP; (iii) closest genes to other types of SNPs in very high LD (r^2>0.80) with the corresponding gSNP, and; (iv) eQTL gene names (including all significant eQTL genes from BRAINEAC,[24] Westra et al,[23] and GTEx brain.[25] This merged query gene set was then used to build a composite functional association network using the

GeneMANIA algorithm together with its large set of accompanying functional association data.[26] We used the Cytoscape software platform,[27] extended by the GeneMANIA plugin (Data Version: July 13, 2017 accessed February 17, 2018).[28] All the genes in the composite network, either from the query or the resulting gene sets, were then used for functional enrichment analysis against Gene Ontology terms (GO terms)[29] to identify the most relevant GO terms using the same plugin.[28] In further sensitivity analyses, we only included genes that were closest to the gSNPs (criterion i), or had strong functional evidence as based on LD of gSNPs with coding SNPs (criterion ii) and/or eQTLs (criterion iv).

Since the abovementioned functional enrichment analysis is biased towards well-characterized genes, we also conducted gene prioritization, gene-set enrichment and tissue/cell type enrichment analyses using DEPICT, which also performs functional prediction for uncharacterized genes according to co-expression data. Our analyses were based on our four bivariate GWAS results. We applied DEPICT default settings with a p-value threshold of 1×10^{-5}, r^2 of 0.05 and physical distance of 500 kb.[30]

Selection of genes from our 13 replicated GWAS loci that are likely to be functional based on location (nearest genes), genes with nsSNPs in LD with the gSNPs and eQTL genes.

BAG5: encodes a protein in the BAG family. BAG5 enhances dopaminergic neuron degeneration and it is involved in Parkinson's disease.[31, 32]

C4A: This gene is located in the MHC class III region and encodes the complement factor 4 (Rodgers blood group). It has a higher expression in liver and adrenal tissue; however, it is also localized to neuronal synapses, dendrites, axons, and cell bodies. An elevated copy number of *C4* genes and/or increased C4 protein expression is observed in Alzheimer's disease (AD) and schizophrenia (SCZ) patients as compared with controls, highlighting a possible role for C4 in the risk of developing AD and providing an explanation for the reduced number of synapses in SCZ, given its intermediary role in synapse elimination.[33-36]

FHIT is a member of the histidine triad gene family, involved in purine metabolism.[31] Polymorphisms of this gene were associated with carcinomas and depressive symptoms.[3, 31]

GNL3: encodes guanine nucleotide-binding protein-like 3 which is thought to interact with p53 and appears to be involved in ribosome biogenesis, cell proliferation and tumorigenesis. The gene variations are reported to be associated with autism spectrum disorder (ASD), SCZ and bipolar disorder.[6, 7, 37]

ITIH1, ***ITIH3***, ***ITIH4*** are a family of genes on chromosome 3 that encode for plasma proteins collectively referred to as the heavy chain subunit of the pre-alpha-trypsin inhibitor complex.[31] The protein complex is involved in events such as gene transcription, precursor processing, and changes in plasma level of other plasma proteins such as hyaluronan and ligands.

KLC1: encodes a tetrameric molecule called conventional kinesin that is composed of two heavy chains and two light chains. These chains are thought to be involved in binding of cargos such as vesicles, mitochondria, and the Golgi complex. KLC1 variant E was highly expressed in the in brain and lymphocytes of patients with AD.[38]

LINC01360: encodes long intergenic non-protein coding RNA 1360 with a biased expression towards testis and choroid plexus. It is observed to be upregulated with alcohol exposure and like many other lncRNAs little is known about its specific function. Variants in the gene are associated with ASD, SCZ, MDD and body mass index.[37, 39-43]

https://www.ebi.ac.uk/gxa/home & https://www.genenames.org/

LINC01929: encodes a long non-protein coding RNA which affects the expression level of some genes in blood monocytes and its polymorphisms are associated with MDD and AD.[44-46]

LOC102546299: encodes 16 transcript variants of type antisense RNA. It is highly expressed in testis and in caudate nucleus, putamen and pituitary gland as well. It also seems to be upregulated against alcohol exposure and variants of this gene are associated with nicotine dependence, AD and MDD. It is found to have effects on methylation levels for *RBM9* (regulator of alternative splicing in the nervous system) and *MED12* (a part of transcription preinitiation complex) in temporal cortex and on expression levels of several genes in blood monocytes.[34, 41, 47, 48, 49]

MIR3143: encodes microRNA 3143 of which its variants are associated with ASD or SCZ.[37, 50]

MUC21: encodes a large membrane-bound glycoprotein of the mucin family, which play an important role in forming mucous layers on epithelial surfaces. These proteins are also

involved in intracellular signalling. Variants of this gene are reported to be associated with ASD or SCZ.[37, 51]

NEK4: encodes NIMA related kinase 4, a serine/threonine kinase required for normal cell senescence and is necessary for cell cycle arrest in response to DNA damage. It also plays a role in maintaining cilium integrity, and polymorphisms in this gene have been associated with ASD, SCZ and bipolar disorder (BPD).[37, 52, 53]

PRSS16: encodes a serine protease that highly expressed in the thymus, and thought to play a role in the antigen presenting pathway. Common variants on this gene were associated with Schizophrenia and ZNF804a regulates expression of the *PRSS16* gene.[54]

PSORS1C2: Psoriasis susceptibility 1 candidate 2 gene has a restricted expression in skin and is reported to be involved in keratinocyte differentiation and psoriasis implications. Little is known about its function, but its variants are reported to be associated with ASD and SCZ.[34, 37, 55, 56]

RTN1**:** is a member of a family of genes that encode reticulons proteins, membrane proteins shaping the tubular endoplasmic reticulum.[31, 57] These proteins are involved in neuroendocrine secretion and in membrane trafficking in neuroendocrine cells. They have been implicated in the onset of neurodegenerative disorders.[58]

SKIV2L: is located in the MHC class III region and encodes Ski2 like RNA helicase which is known as a DEAD box protein characterized by the conserved motif Asp-Glu-Ala-Asp (DEAD). DEAD-box proteins are involved in a number of cellular processes altering RNA secondary structure such as translation initiation, splicing, and ribosome and spliceosome assembly. This gene is previously reported to be associated with neuroblastoma, ASD and SCZ.[37, 59-61]

SPATA31D1: encodes SPATA31 subfamily D member 1 and little is known about its exact function. However, it is mainly expressed in testis and is classified as a part of spermatogenesis and cell differentiation processes. There are also reports on its expression in other tissues such as blood. Variation of the gene is reported to be associated with ASD or SCZ.[34, 37, 62]

SPCS1: encodes signal peptidase complex subunit 1, located within the endoplasmic reticulum membrane and involved in metabolism and targeting of proteins such as peptide hormones. Its downregulation is observed in brain areas of AD patients including hippocampus and cortex. Variants in this gene are reported to be associated with ASD, SCZ and BPD.[6, 37, 53, 63]

STAB1: encodes stabilin 1, a large transmembrane receptor protein involved in inflammation, defense response to bacterium and negative regulation of angiogenesis. The expression of stabilin 1 is biased towards spleen and placenta and it is differentially expressed in blood samples of bipolar disorder (BPD) cases vs controls. Variants in the gene are reported to be associated with ASD, SCZ, BPD, cognitive function and Parkinson's disease.[6, 34, 37, 53, 64-67]

STK19: is located in the major histocompatibility complex (MHC) class III region on chromosome 6. It encodes a serine/threonine kinase that localizes predominantly to the nucleus. Although its specific function is unknown, it is possible that phosphorylation of this protein is involved in transcriptional regulation.[31]

***TCF4*:** encodes transcription factor 4, a basic helix-loop-helix transcription factor. It is broadly expressed in the brain and plays an important role in the nervous system development and in the regulation of neuron differentiation.[31]

XRCC3: encodes X-ray repair cross-complementing 3, a member of the RecA/Rad51-related protein family which play a role in homologous recombination to repair DNA damage and maintain chromosome stability. It has been involved in a number of cancers and its variants are reported to be associated with ASD and SCZ.[37, 66, 68, 69]

ZNF804A: encodes a zinc finger binding protein. Zinc finger proteins are functionally involved in DNA recognition, RNA packaging, transcriptional activation, regulation of apoptosis, protein folding and assembly, and lipid binding. Genetic variation in the ZNF804A gene has shown an effect on the thickness of the prefrontal and temporal cortex and on white matter microstructure in patients with schizophrenia.[31, 70]

References

1. Hek K, Demirkan A, Lahti J, Terracciano A, Teumer A, Cornelis MC *et al.* A genome-wide association study of depressive symptoms. *Biological Psychiatry* 2013; **73**(7)**:** 667--678.

2. Sullivan PF, Daly MJ, Ripke S, Lewis CM, Lin D-Y, Wray NR *et al.* A mega-analysis of genome-wide association studies for major depressive disorder. *Molecular Psychiatry* 2013; **18**(4)**:** 497--511.

3. Direk N, Williams S, Smith JA, Ripke S, Air T, Amare AT *et al.* An Analysis of Two Genome-Wide Association Meta-Analyses Identifies a New Locus for Broad Depression Phenotype. *Biological Psychiatry* 2016**:** 1--23.

4. Hyde CL, Nagle MW, Tian C, Chen X, Paciga SA, Wendland JR *et al.* Identification of 15 genetic loci associated with risk of major depression in individuals of European descent. *Nature Genetics* 2016; **48**(9)**:** 1031--1036.

5. CONVERGE Consortium. Sparse whole-genome sequencing identifies two loci for major depressive disorder. *Nature* 2015; **523**(7562)**:** 588.

6. Psychiatric GWAS Consortium Bipolar Disorder Working Group. Large-scale genome-wide association analysis of bipolar disorder identifies a new susceptibility locus near ODZ4. *Nat Genet* 2011; **43**(10)**:** 977-983.

7. Ripke S, Neale BM, Corvin A, Walters JTR, Farh K-H, Holmans Pa *et al.* Biological insights from 108 schizophrenia-associated genetic loci. *Nature* 2014; **511**(7510)**:** 421--427.

8. Frazer KA, Ballinger DG, Cox DR, Hinds DA, Stuve LL, Gibbs RA *et al.* A second generation human haplotype map of over 3.1 million SNPs. *Nature* 2007; **449**(7164)**:** 851--861.

9. Abecasis GR, Altshuler D, Auton A, Brooks LD, Durbin RM, Gibbs RA *et al.* A map of human genome variation from population-scale sequencing. *Nature* 2010; **467**(7319)**:** 1061--1073.

10. Fritsche LG, Igl W, Bailey JN, Grassmann F, Sengupta S, Bragg-Gresham JL *et al.* A large genome-wide association study of age-related macular degeneration highlights contributions of rare and common variants. *Nat Genet* 2016; **48**(2)**:** 134-143.

11. Zheng HF, Forgetta V, Hsu YH, Estrada K, Rosello-Diez A, Leo PJ *et al.* Whole-genome sequencing identifies EN1 as a determinant of bone density and fracture. *Nature* 2015; **526**(7571)**:** 112-117.

12. Millard LAC, Davies NM, Gaunt TR, Davey Smith G, Tilling K. Software Application Profile: PHESANT: a tool for performing automated phenome scans in UK Biobank. *Int J Epidemiol* 2017.

13. Vaez A, Jansen R, Prins BP, Hottenga J-JJa. In Silico Post Genome-Wide Association Studies Analysis of C-Reactive Protein Loci Suggests an Important Role for Interferons. *Circulation: Cardiovascular Genetics* 2015; **8**(3)**:** 487--497.

14. Okbay A, Baselmans BM, De Neve JE, Turley P, Nivard MG, Fontana MA *et al.* Genetic variants associated with subjective well-being, depressive symptoms, and neuroticism identified through genome-wide analyses. *Nat Genet* 2016; **48**(6)**:** 624-633.

15. Wray NR, Ripke S, Mattheisen M, Trzaskowski M, Byrne EM, Abdellaoui A *et al.* Genome-wide association analyses identify 44 risk variants and refine the genetic architecture of major depression. *Nature Genetics* 2018; **50**(5)**:** 668-681.

16. Welter D, MacArthur J, Morales J, Burdett T, Hall P, Junkins H *et al.* The NHGRI GWAS Catalog, a curated resource of SNP-trait associations. *Nucleic Acids Research* 2014; **42**(Database issue)**:** D1001--1006.

17. Danecek P, Auton A, Abecasis G, Albers CA, Banks E, DePristo MA *et al.* The variant call format and VCFtools. *Bioinformatics* 2011; **27**(15)**:** 2156--2158.

18. Li H. Tabix: fast retrieval of sequence features from generic TAB-delimited files. *Bioinformatics* 2011; **27**(5)**:** 718--719.

19. Purcell S, Neale B, Todd-Brown K, Thomas L, Ferreira MARR, Bender D *et al.* PLINK: A tool set for whole-genome association and population-based linkage analyses. *American Journal of Human Genetics* 2007; **81**(3)**:** 559--575.

20. Wang K, Li M, Hakonarson H. ANNOVAR: functional annotation of genetic variants from high-throughput sequencing data. *Nucleic Acids Research* 2010; **38**(16)**:** e164--e164.

21. Kumar P, Henikoff S, Ng PC. Predicting the effects of coding non-synonymous variants on protein function using the SIFT algorithm. *Nature Protocols* 2009; **4**(8)**:** 1073--1081.

22. Adzhubei IA, Schmidt S, Peshkin L, Ramensky VE, Gerasimova A, Bork P *et al.* A method and server for predicting damaging missense mutations. *Nature Methods* 2010; **7**(4)**:** 248--249.

23. Westra H-J, Peters MJ, Esko T, Yaghootkar H, Schurmann C, Kettunen J *et al.* Systematic identification of trans eQTLs as putative drivers of known disease associations. *Nature Genetics* 2013; **45**(10)**:** 1238--1243.

24. Ramasamy A, Trabzuni D, Guelfi S, Varghese V, Smith C, Walker R *et al.* Genetic variability in the regulation of gene expression in ten regions of the human brain. *Nature Neuroscience* 2014; **17**(10)**:** 1418--1428.

25. GTEx Consortium. The Genotype-Tissue Expression (GTEx) pilot analysis: Multitissue gene regulation in humans. *Science* 2015; **348**(6235)**:** 648--660.

26. Mostafavi S, Ray D, Warde-Farley D, Grouios C, Morris Q. GeneMANIA: a real-time multiple association network integration algorithm for predicting gene function. *Genome Biology* 2008; **9**(Suppl 1)**:** S4.

27. Saito R, Smoot ME, Ono K, Ruscheinski J, Wang P-L, Lotia S *et al.* A travel guide to Cytoscape plugins. *Nature Methods* 2012; **9**(11)**:** 1069--1076.

28. Montojo J, Zuberi K, Rodriguez H, Kazi F, Wright G, Donaldson SL *et al.* GeneMANIA Cytoscape plugin: fast gene function predictions on the desktop. *Bioinformatics* 2010; **26**(22)**:** 2927--2928.

29. Ashburner M, Ball CA, Blake JA, Botstein D, Butler H, Cherry JM *et al.* Gene Ontology: tool for the unification of biology. *Nature Genetics* 2000; **25**(1)**:** 25--29.

30. Pers TH, Karjalainen JM, Chan Y, Westra H-J, Wood AR, Yang J *et al.* Biological interpretation of genome-wide association studies using predicted gene functions. *Nature Communications* 2015; **6:** 5890.

31. Pruitt K, Brown G, Tatusova T, Maglott D. The Reference Sequence (RefSeq) Database. 2012.

32. Kalia SK, Lee S, Smith PD, Liu L, Crocker SJ, Thorarinsdottir TE *et al.* BAG5 inhibits parkin and enhances dopaminergic neuron degeneration. *Neuron* 2004; **44**(6)**:** 931--945.

33. Sekar A, Bialas AR, de Rivera H, Davis A, Hammond TR, Kamitaki N *et al.* Schizophrenia risk from complex variation of complement component 4. *Nature* 2016; **530**(7589)**:** 177-183.

34. Fagerberg L, Hallstrom BM, Oksvold P, Kampf C, Djureinovic D, Odeberg J *et al.* Analysis of the human tissue-specific expression by genome-wide integration of transcriptomics and antibody-based proteomics. *Molecular & Cellular Proteomics: MCP* 2014; **13**(2)**:** 397-406.

35. Zorzetto M, Datturi F, Divizia L, Pistono C, Campo I, De Silvestri A *et al.* Complement C4A and C4B Gene Copy Number Study in Alzheimer's Disease Patients. *Current Alzheimer Research* 2017; **14**(3)**:** 303-308.

36. Pruitt KD, Brown GR, Hiatt SM, Thibaud-Nissen F, Astashyn A, Ermolaeva O *et al.* RefSeq: an update on mammalian reference sequences. *Nucleic Acids Res* 2014; **42**(Database issue)**:** D756-763.

37. Autism Spectrum Disorders Working Group of The Psychiatric Genomics C. Meta-analysis of GWAS of over 16,000 individuals with autism spectrum disorder highlights a novel locus at 10q24.32 and a significant overlap with schizophrenia. *Molecular Autism* 2017; **8:** 21.

38. Morihara T, Hayashi N, Yokokoji M, Akatsu H, Silverman MA, Kimura N *et al.* Transcriptome analysis of distinct mouse strains reveals kinesin light chain-1 splicing as an amyloid-β accumulation modifier. *Proceedings of the National Academy of Sciences of the United States of America* 2014; **111**(7)**:** 2638--2643.

39. Akiyama M, Okada Y, Kanai M, Takahashi A, Momozawa Y, Ikeda M *et al.* Genome-wide association study identifies 112 new loci for body mass index in the Japanese population. *Nat Genet* 2017; **49**(10)**:** 1458-1467.

40. Shyn SI, Shi J, Kraft JB, Potash JB, Knowles JA, Weissman MM *et al.* Novel loci for major depression identified by genome-wide association study of Sequenced Treatment Alternatives to Relieve Depression and meta-analysis of three studies. *Mol Psychiatry* 2011; **16**(2)**:** 202-215.

41. Kupfer DM, White VL, Strayer DL, Crouch DJ, Burian D. Microarray characterization of gene expression changes in blood during acute ethanol exposure. *BMC Medical Genomics* 2013; **6:** 26.

42. Petryszak R, Keays M, Tang YA, Fonseca NA, Barrera E, Burdett T *et al.* Expression Atlas update--an integrated database of gene and protein expression in humans, animals and plants. *Nucleic Acids Research* 2016; **44**(D1)**:** D746-752.

43. Yates B, Braschi B, Gray KA, Seal RL, Tweedie S, Bruford EA. Genenames.org: the HGNC and VGNC resources in 2017. *Nucleic Acids Research* 2017; **45**(D1)**:** D619-d625.

44. Hu X, Pickering E, Liu YC, Hall S, Fournier H, Katz E *et al.* Meta-analysis for genome-wide association study identifies multiple variants at the BIN1 locus associated with late-onset Alzheimer's disease. *PLoS One* 2011; **6**(2)**:** e16616.

45. Zeller T, Wild P, Szymczak S, Rotival M, Schillert A, Castagne R *et al.* Genetics and beyond--the transcriptome of human monocytes and disease susceptibility. *PLoS One* 2010; **5**(5)**:** e10693.

46. Wray NR, Pergadia ML, Blackwood DH, Penninx BW, Gordon SD, Nyholt DR *et al.* Genome-wide association study of major depressive disorder: new results, meta-analysis, and lessons learned. *Mol Psychiatry* 2012; **17**(1)**:** 36-48.

47. Harold D, Abraham R, Hollingworth P, Sims R, Gerrish A, Hamshere ML *et al.* Genome-wide association study identifies variants at CLU and PICALM associated with Alzheimer's disease. *Nat Genet* 2009; **41**(10)**:** 1088-1093.

48. Shi J, Potash JB, Knowles JA, Weissman MM, Coryell W, Scheftner WA *et al.* Genome-wide association study of recurrent early-onset major depressive disorder. *Mol Psychiatry* 2011; **16**(2)**:** 193-201.

49. Hon CC, Ramilowski JA, Harshbarger J, Bertin N, Rackham OJ, Gough J *et al.* An atlas of human long non-coding RNAs with accurate 5' ends. *Nature* 2017; **543**(7644)**:** 199-204.

50. Pruitt KD, Tatusova T, Klimke W, Maglott DR. NCBI Reference Sequences: current status, policy and new initiatives. *Nucleic Acids Res* 2009; **37**(Database issue)**:** D32-36.

51. O'Leary NA, Wright MW, Brister JR, Ciufo S, Haddad D, McVeigh R *et al.* Reference sequence (RefSeq) database at NCBI: current status, taxonomic expansion, and functional annotation. *Nucleic Acids Research* 2016; **44**(D1)**:** D733-745.

52. Scott LJ, Muglia P, Kong XQ, Guan W, Flickinger M, Upmanyu R *et al.* Genome-wide association and meta-analysis of bipolar disorder in individuals of European ancestry. *Proc Natl Acad Sci U S A* 2009; **106**(18)**:** 7501-7506.

53. Ripke S, O'Dushlaine C, Chambert K, Moran JL, Kahler AK, Akterin S *et al.* Genome-wide association analysis identifies 13 new risk loci for schizophrenia. *Nat Genet* 2013; **45**(10)**:** 1150-1159.

54. Girgenti MJ, LoTurco JJ, Maher BJ, Schaefer CA, Wyatt RJ. ZNF804a Regulates Expression of the Schizophrenia-Associated Genes PRSS16, COMT, PDE4B, and DRD2. *PLoS One* 2012; **7**(2)**:** e32404.

55. Abbas Zadeh S, Mlitz V, Lachner J, Golabi B, Mildner M, Pammer J *et al.* Phylogenetic profiling and gene expression studies implicate a primary role of PSORS1C2 in terminal differentiation of keratinocytes. *Experimental Dermatology* 2017; **26**(4)**:** 352-358.

56. Holm SJ, Carlen LM, Mallbris L, Stahle-Backdahl M, O'Brien KP. Polymorphisms in the SEEK1 and SPR1 genes on 6p21.3 associate with psoriasis in the Swedish population. *Experimental Dermatology* 2003; **12**(4)**:** 435-444.

57. Voeltz GK, Prinz Wa, Shibata Y, Rist JM, Rapoport Ta. A class of membrane proteins shaping the tubular endoplasmic reticulum. *Cell* 2006; **124**(3)**:** 573--586.

58. Yan R, Shi Q, Hu X, Zhou X. Reticulon proteins: emerging players in neurodegenerative diseases. *Cellular and Molecular Life Sciences: CMLS* 2006; **63**(7-8)**:** 877--889.

59. Capasso M, Diskin SJ, Totaro F, Longo L, De Mariano M, Russo R *et al.* Replication of GWAS-identified neuroblastoma risk loci strengthens the role of BARD1 and affirms the cumulative effect of genetic variations on disease susceptibility. *Carcinogenesis* 2013; **34**(3)**:** 605-611.

60. van der Valk RJ, Kreiner-Moller E, Kooijman MN, Guxens M, Stergiakouli E, Saaf A *et al.* A novel common variant in DCST2 is associated with length in early life and height in adulthood. *Hum Mol Genet* 2015; **24**(4)**:** 1155-1168.

61. Kopplin LJ, Igo RP, Jr., Wang Y, Sivakumaran TA, Hagstrom SA, Peachey NS *et al.* Genome-wide association identifies SKIV2L and MYRIP as protective factors for age-related macular degeneration. *Genes and Immunity* 2010; **11**(8)**:** 609-621.

62. Bairoch A, Cohen-Boulakia S, Froidevaux C, (eds). Bgee: Integrating and Comparing Heterogeneous Transcriptome Data Among Species. *Proceedings of the Data Integration in the Life Sciences*; 2008// 2008; Berlin, Heidelberg. Springer Berlin Heidelberg.

63. Kalies KU, Hartmann E. Membrane topology of the 12- and the 25-kDa subunits of the mammalian signal peptidase complex. *The Journal of Biological Chemistry* 1996; **271**(7)**:** 3925-3929.

64. Davies G, Armstrong N, Bis JC, Bressler J, Chouraki V, Giddaluru S *et al.* Genetic contributions to variation in general cognitive function: a meta-analysis of genome-wide association studies in the CHARGE consortium (N=53949). *Mol Psychiatry* 2015; **20**(2)**:** 183-192.

65. Chang D, Nalls MA, Hallgrimsdottir IB, Hunkapiller J, van der Brug M, Cai F *et al.* A meta-analysis of genome-wide association studies identifies 17 new Parkinson's disease risk loci. *Nat Genet* 2017; **49**(10)**:** 1511-1516.

66. Schizophrenia Working Group of the Psychiatric Genomics Consortium. Biological insights from 108 schizophrenia-associated genetic loci. *Nature* 2014; **511**(7510)**:** 421-427.

67. Witt SH, Juraeva D, Sticht C, Strohmaier J, Meier S, Treutlein J *et al.* Investigation of manic and euthymic episodes identifies state- and trait-specific gene expression and STAB1 as a new candidate gene for bipolar disorder. *Translational Psychiatry* 2014; **4:** e426.

68. Ali AM, AbdulKareem H, Al Anazi M, Reddy Parine N, Shaik JP, Alamri A *et al.* Polymorphisms in DNA Repair Gene XRCC3 and Susceptibility to Breast Cancer in Saudi Females. *BioMed Research International* 2016; **2016:** 8721052.

69. He Y, Penney ME, Negandhi AA, Parfrey PS, Savas S, Yilmaz YE. XRCC3 Thr241Met and TYMS variable number tandem repeat polymorphisms are associated with time-to-metastasis in colorectal cancer. *PLoS One* 2018; **13**(2)**:** e0192316.

70. Ikuta T, Peters BD, Guha S, John M, Karlsgodt KH, Lencz T *et al.* A schizophrenia risk gene, ZNF804A, is associated with brain white matter microstructure. *Schizophrenia Research* 2014; **155**(1-3)**:** 15--20.
