# Supplementary material for: Bivariate genome-wide association analyses of the broad depression phenotype combined with major depressive disorder, bipolar disorder or schizophrenia reveal eight novel genetic loci for depression
Source: Mol Psychiatry. 2019 Jan 9;25(7):1420–9. doi: 10.1038/s41380-018-0336-6 (PMC7303007; doi:10.1038/s41380-018-0336-6)
Supplement: Supplementary file 3 — Supplementary Table 1 [file 41380_2018_336_MOESM3_ESM.pdf]

Supplementary Table 1: Replication results in the UK Biobank for all three-depression outcomes (broad depression, probable MDD and ICD-coded MDD)[1] for the 22 genome-wide significant SNPs from bivariate discovery meta-analyses.

							Broad depression			Probable MDD			ICD-coded MDD		
**gSNP**	**A1**	**A2**	**Chr**	**Position/ Ch37/hg19**	**EAF**	**Imputation accuracy**	**Beta**	**SE**	**P-value (2-sided)**	**Beta**	**SE**	**P-value (2-sided)**	**Beta**	**SE**	**P-value (2-sided)**
rs12407717	T	C	1	30426014	0.13	0.998	-0.0016	0.0018	3.64x10^{-1}	-0.0022	0.0019	2.47x10^{-1}	-7.29 x10^{-4}	8.71 x10^{-4}	4.03x10^{-1}
rs12128108	T	C	1	50293421	0.23	1.000	0.0021	0.0014	1.33x10^{-1}	0.0010	0.0015	5.07x10^{-1}	5.37 x10^{-4}	6.91 x10^{-4}	4.37x10^{-1}
rs2422320	T	C	1	73293332	0.43	0.997	-0.0048	0.0012	**7.32x10^{-5}**	-0.0028	0.0013	**3.11x10^{-2}**	-0.0010	5.92 x10^{-4}	8.81x10^{-2}
rs2318763	A	G	1	150115974	0.17	0.999	0.0015	0.0016	3.37x10^{-1}	0.0027	0.0017	1.10x10^{-1}	0.0010	7.77 x10^{-4}	1.91x10^{-1}
rs16836940	A	G	1	150416913	0.22	1.000	7.64x10^{-4}	0.0014	5.91x10^{-1}	4.66x10^{-4}	0.0016	7.64x10^{-1}	1.22 x10^{-4}	7.01 x10^{-4}	8.62x10^{-1}
rs7597593	T	C	2	185533580	0.62	0.998	0.0025	0.0012	**4.28x10^{-2}**	0.0024	0.0013	7.05x10^{-2}	0.0013	6.00 x10^{-4}	**2.58x10^{-2}**
rs2535629	A	G	3	52833219	0.33	1.000	-0.0031	0.0013	**1.32x10^{-2}**	-0.0008	0.0014	5.79x10^{-1}	-7.23 x10^{-4}	6.18 x10^{-4}	2.43x10^{-1}
rs1966136	A	C	3	61153578	0.41	1.000	-0.0039	0.0012	**1.29x10^{-3}**	-0.0004	0.0013	7.87x10^{-1}	-4.47 x10^{-4}	5.91 x10^{-4}	4.49x10^{-1}
rs7714851	T	C	5	164475774	0.52	1.000	-0.0057	0.0012	**1.07x10^{-6}**	-0.0021	0.0013	1.03x10^{-1}	-0.0014	5.82 x10^{-4}	**1.31x10^{-2}**
rs911186	A	G	6	27150599	0.23	1.000	0.0040	0.0014	**3.99x10^{-3}**	0.0019	0.0015	2.18x10^{-1}	7.56 x10^{-4}	6.92 x10^{-4}	2.75x10^{-1}
rs2894052[A]	C	G	6	30938128	0.14	0.999	0.0044	0.0017	**9.50x10^{-3}**	0.0029	0.0019	1.18x10^{-1}	0.0019	0.0008	**2.48x10^{-2}**
rs1265099	A	G	6	31105413	0.41	1.000	0.0030	0.0012	**1.38x10^{-2}**	8.06x10^{-4}	0.0013	5.40x10^{-1}	5.21 x10^{-4}	5.93 x10^{-4}	3.79x10^{-1}
rs389883	T	G	6	31947460	0.68	1.000	0.0051	0.0013	**4.73x10^{-5}**	0.0026	0.0014	5.91x10^{-2}	0.0016	6.23 x10^{-4}	**1.00x10^{-2}**
rs4976976	A	G	8	143311653	0.40	0.999	-0.0016	0.0012	1.85x10^{-1}	-0.0016	0.0013	2.31x10^{-1}	-6.31 x10^{-4}	5.94 x10^{-4}	2.88x10^{-1}
rs2026194	T	C	9	85082899	0.24	1.000	0.0037	0.0014	**7.24x10^{-3}**	0.0029	0.0015	5.46x10^{-2}	1.89 x10^{-4}	6.76 x10^{-4}	7.80x10^{-1}
rs4765914	T	C	12	2420377	0.79	0.997	0.0025	0.0014	8.48x10^{-2}	0.0012	0.0016	4.35x10^{-1}	-2.94 x10^{-4}	7.16 x10^{-4}	6.82x10^{-1}
rs10774037	A	G	12	2420526	0.79	0.997	-0.0025	0.0015	8.95x10^{-2}	-0.0014	0.0016	3.91x10^{-1}	2.80 x10^{-4}	7.17 x10^{-4}	6.96x10^{-1}
rs2182139	T	C	14	60149233	0.75	1.000	0.0040	0.0014	**3.71x10^{-3}**	0.0010	0.0015	4.94x10^{-1}	4.88 x10^{-4}	6.74 x10^{-4}	4.70x10^{-1}
rs9323497	T	C	14	67873128	0.05	0.998	5.96x10^{-4}	0.0027	8.24x10^{-1}	-0.0014	0.0029	6.30x10^{-1}	-0.0016	0.0013	2.21x10^{-1}
rs4906335	A	C	14	104021141	0.28	0.998	0.0042	0.0013	**1.45x10^{-3}**	0.0033	0.0014	**2.18x10^{-2}**	-5.96 x10^{-5}	6.51 x10^{-4}	9.27x10^{-1}
rs4843613	T	C	16	87489698	0.83	0.999	0.0026	0.0016	8.96x10^{-2}	-0.0011	0.0017	5.25x10^{-1}	-4.91 x10^{-4}	7.68 x10^{-4}	5.22x10^{-1}
rs9951150	A	G	18	52821124	0.46	0.999	-0.0031	0.0012	**9.27x10^{-3}**	-5.46x10^{-4}	0.0013	6.74x10^{-1}	-3.77 x10^{-4}	5.85 x10^{-4}	5.19x10^{-1}

Abbreviations: A1, effect allele; A2, other allele; EAF, effect allele frequency

Replication p-values < 0.05 are **bolded**. Beta effect sizes and standard errors are reported on the 0-1 scale

[A] Proxy for rs9368649 with r^2=1
