# Supplementary material for: Bivariate genome-wide association analyses of the broad depression phenotype combined with major depressive disorder, bipolar disorder or schizophrenia reveal eight novel genetic loci for depression
Source: Mol Psychiatry. 2019 Jan 9;25(7):1420–9. doi: 10.1038/s41380-018-0336-6 (PMC7303007; doi:10.1038/s41380-018-0336-6)
Supplement: Supplementary file 4 — Supplementary Table 2 [file 41380_2018_336_MOESM4_ESM.pdf]

Supplementary Table 2: Table showing genetic correlation and genome-wide significant bivariate GWAS results of broad depression with outcomes serving as negative controls: 1) age-related macular degeneration; 2) osteoporosis measured as bone mineral density of (i) femoral neck; (ii) lumbar spine and; (iii) forearm; 3) cancer of (i) breast and; (ii) prostate.

SNP	Chr	Position	Bivariate GWAS results		
			**A1**	**A2**	**Bivariate P-value**
Age-related macular degeneration[1] & broad depression[2] (r_g=0.104, p=0.37)					
rs17651057	10	124531450	G	A	2.72×10^{-8}
Osteoporosis (bone mineral density)					
(i) Femoral Neck[3] & broad depression[2] (r_g=-0.013, p=0.96)					
(ii) Lumbar Spine[3] & broad depression[2] (r_g=-0.070, p=0.33)					
(iii) Forearm[3] & broad depression[2] (r_g=-0.031, p=0.92)					
rs7714851	5	164475774	T	C	1.29×10^{-10}
rs1483582	8	116480753	G	A	2.67×10^{-8}
Breast cancer UKB & broad depression[2] (r_g=0.10, p=0.10)					
rs11208611	1	65684425	C	T	4.22×10^{-8}
rs10888582	1	150428819	G	A	4.16×10^{-9}
Prostate cancer UKB & broad depression[2] (r_g=0.054, p=0.93)					
rs12477089	2	242265313	C	A	1.52×10^{-9}
rs2439644	8	96226764	C	T	7.01×10^{-9}

Abbreviations: r_g: genetic correlation

Source of data: UKB: UK biobank data

https://docs.google.com/spreadsheets/d/1b3oGI2lUt57BcuHttWaZotQcI0-

mBRPyZihz87Ms_No/edit#gid=1209628142

Reference

1. Fritsche LG, Igl W, Bailey JN, Grassmann F, Sengupta S, Bragg-Gresham JL *et al.* A large genome-wide association study of age-related macular degeneration highlights contributions of rare and common variants. *Nat Genet* 2016; **48**(2)**:** 134-143.

2. Direk N, Williams S, Smith JA, Ripke S, Air T, Amare AT *et al.* An Analysis of Two Genome-wide Association Meta-analyses Identifies a New Locus for Broad Depression Phenotype. *Biol Psychiatry* 2017; **82**(5)**:** 322-329.

3. Zheng HF, Forgetta V, Hsu YH, Estrada K, Rosello-Diez A, Leo PJ *et al.* Whole-genome sequencing identifies EN1 as a determinant of bone density and fracture. *Nature* 2015; **526**(7571)**:** 112-117.
