# Supplementary material for: Bivariate genome-wide association analyses of the broad depression phenotype combined with major depressive disorder, bipolar disorder or schizophrenia reveal eight novel genetic loci for depression
Source: Mol Psychiatry. 2019 Jan 9;25(7):1420–9. doi: 10.1038/s41380-018-0336-6 (PMC7303007; doi:10.1038/s41380-018-0336-6)
Supplement: Supplementary file 6 — Supplementary Table 4 [file 41380_2018_336_MOESM6_ESM.pdf]

Supplementary Table 4: eQTL analysis results for all 56 gSNPs. Genes with eQTLs in multiple databases in bold.

SNP	CHR	BP	Allele	eQTL lookup in three databases		
				Westra et al, FDR<0.05	BRAINEAC, $p<1\times10^{-5}$	GTEx, FDR<0.05
rs301806	1	8482078	C/T	***RERE***	***RERE***	
rs1432639	1	72813218	C/A			*NEGR1, RPL31P12*
rs9427672	1	197754741	A/G		*DENND1B*	
rs2535629	3	52833219	G/A	***ITIH4***, *SPCS1*	***ITIH4***,***GNL3***, *GLT8D1, SNORD19B*	***ITIH4***, ***GNL3***, *PPM1M*
rs7430565	3	158107180	G/A		*RARRES1, MLF1*	
rs34215985	4	42047778	C/G			*SLC30A9*
rs10514299	5	87663610	C/T			*TMEM161B-AS1*
rs454214	5	88003403	C/T	*MEF2C*		*CTC-467M3.3*
rs911186	6	27150599	A/G	*HIST1H2BK*, ***PRSS16***	*ZNF389, BTN2A1,BTN2A2*	*ZNF204P*, ***PRSS16***
rs115507122	6	30737591	G/C		*VARS2*, ***MICB***	***MICB***, *ATAT1, FLOT1*
rs9368649	6	30938883	A/G	*VARSL*		
rs1265099	6	31105413	A/G	*HCG27*	*PSORS1C2*	*C4A*
rs389883	6	31947460	G/T	***SKIV2L***, *HSPA1B, DOM3Z*	*NFKBIL1*	*HLA-DRB1, HLA-DRB5, CYP21A1P*, ***SKIV2L***, *HLA-C, WASF5P, XXbac-BPG248L24.12*
rs1475120	6	105389953	G/A			*LINC00577*
rs7029033	9	126682068	C/T		*LHX2*	
rs12552	13	53625781	A/G	*OLFM4*		
rs4904738	14	42179732	T/C		***LRFN5***	***LRFN5***

rs2182139	14	60149233	T/C	*RTN1, C14orf100*		
rs915057	14	64686207	A/G		*SYNE2*	
rs4906335	14	104021141	C/A	*BAG5, CKB, EIF5,* ***XRCC3***,***KLC1***	***KLC1***,*C14orf153*	***XRCC3***
rs11643192	16	72214276	C/A	*HP*		
rs1833288	18	52517906	A/G		*RAB27B*	
rs2179744	22	41621714	G/A	*EP300, L3MBTL2, CHADL, TEF, RANGAP1*	*XPNPEP3*	
