# Supplementary material for: Bivariate genome-wide association analyses of the broad depression phenotype combined with major depressive disorder, bipolar disorder or schizophrenia reveal eight novel genetic loci for depression
Source: Mol Psychiatry. 2019 Jan 9;25(7):1420–9. doi: 10.1038/s41380-018-0336-6 (PMC7303007; doi:10.1038/s41380-018-0336-6)
Supplement: Supplementary file 7 — Supplementary Table 5 [file 41380_2018_336_MOESM7_ESM.pdf]

Supplementary Table 5: *In silico* pleiotropy analysis findings that show traits significantly associated with SNPs linked (r^2>0.50) to the 13 gSNPs

gSNP	Linked-SNP	CHR	BP	A1	A2	LD (r^2)	Type	Nearby gene	Pleiotropic effect
**rs2422320**	rs35998080	1	73278615	G	T	1	intergenic	*NEGR1*, *LINC01360*	SCZ, ASD
**rs7597593**	rs7597593	2	185533580	T	C	1	intronic	*ZNF804A*	ASD, ADHD, BPD, MDD, and SCZ
**rs2535629**	rs2535629	3	52833219	G	A	1	intronic	*ITIH3*	ASD,ADHD, BPD, MDD, and SCZ
	rs2240920	3	52831009	C	T	0.92	intronic	*ITIH3*	BMI
	rs736408	3	52835354	C	T	0.89	intronic	*ITIH3*	BPD
	rs4687552	3	52838402	T	C	0.89	intronic	*ITIH3*	SCZ
	rs1042779 [¥]	3	52821011	A	G	0.84	exonic	*ITIH1*	BPD
	rs2300149	3	52822921	C	T	0.8	intronic	*ITIH1*	WCadjBMI
	rs2251219*	3	52584787	T	C	0.76	exonic	*PBRM1*	MDD, BPD
	rs11177[¥]	3	52721305	G	A	0.75	exonic	*GNL3*	Osteoarthritis
	rs11130319	3	52755592	A	T	0.75	intronic	*NEK4*	BMI
	rs10865974	3	52718280	G	T	0.73	intronic	*PBRM1*	BPD
	rs2240919	3	52831701	C	G	0.72	intronic	*ITIH3*	Height
	rs3617 [¥]	3	52833805	C	A	0.64	exonic	*ITIH3*	ASD,SCZ,RBC count

	rs2535627	3	52845105	T	C	0.62	intergenic	*ITIH3,ITIH4*	SCZ
	rs2710323	3	52815905	T	C	0.58	intronic	*ITIH1*	BMI, Glaucoma, SCZ, schizoaffective or BPD
	rs2577831	3	52628056	C	A	0.57	intronic	*PBRM1*	SCZ
	rs2302417	3	52814256	T	A	0.56	intronic	*ITIH1*	BPD
	rs2590838	3	52622086	G	A	0.55	intronic	*PBRM1*	Adiponectin levels
	rs7618915	3	52279594	G	A	0.54	upstream	*PPM1M*	BPD
	rs13083798	3	52649748	A	G	0.54	intronic	*PBRM1*	WHR
	rs1108842	3	52720080	A	C	0.54	UTR5	*GNL3*	Adiponectin levels
	rs2239547	3	52855229	T	C	0.54	intronic	*ITIH4*	SCZ
**rs1966136**	rs2365389	3	61236462	C	T	0.66	intronic	*FHIT*	BMI
	rs911186	6	27150599	A	G	1	intergenic	*MIR3143*	ASD,SCZ
	rs2393911	6	27057079	C	T	0.72	intergenic	*LINC00240, HIST1H2BJ*	Neuroticism
**rs911186**	rs6904071	6	27047256	G	A	0.7	intergenic	*HIST1H2BJ*	Neuroticism
	rs13194053	6	27143883	T	C	0.7	intergenic	*MIR3143, PRSS16*	SCZ
	rs6932590	6	27248931	T	C	0.52	intergenic	*PRSS16, POM121L2*	SCZ
**rs9368649**	rs9368649	6	30938883	A	G	1	intergenic	*DPCR1, MUC21*	Pulmonary function

**rs389883**	rs622871	6	31878495	A	G	0.81	intronic	*C2*	SIA, IMBadjBS, UTI
	rs521977	6	31836827	T	G	0.6	intronic	*SLC44A4*	IMBadjBS, PUM
	rs644045	6	31883957	A	G	0.58	intronic	*C2*	Endometriosis
	rs589428	6	31848220	T	G	0.57	intronic	*EHMT2*	WHRadjBMIns
	rs1313996	6	31876042	T	A	0.57	intronic	*C2*	Shingles
	rs2763981	6	31840021	T	A	0.54	intronic	*SLC44A4*	TIBC
**rs2182139**	rs12431410	14	60166022	C	T	0.6	intronic	*RTN1*	SCZ
**rs915057**	rs1152591	14	64680848	A	G	0.7	intronic	*SYNE2*	Atrial fibrillation
	rs1256061	14	64703593	G	T	0.59	intronic	*ESR2*	Hb concentration,RBC count,Hematocrit
**rs4906335**	rs12887734	14	104046834	G	T	0.92	intronic	*APOPT1*	SCZ
	rs12893668	14	104017793	G	A	0.78	intergenic	*BAG5*	Reticulocyte count
	rs2765041	14	103991478	T	A	0.61	intergenic	*CKB*	High light scatter reticulocyte count
**rs9951150**	rs9951150	18	52821124	A	G	1	intergenic	*LOC101927229,TCF4*	ASD,ADHD, BPD, MDD, and SCZ

¥ Nonsynonymous SNP, *synonymous SNP, gSNPs; genome-wide significant lead SNPs identified in our analyses**.** Abbreviations: SNP: single nucleotide polymorphism, LD: linkage disequilibrium, MDD: major depressive disorder, BPD: bipolar disorder, SCZ: schizophrenia, ASD: autism spectrum disorder, ADHD: attention deficit hyperactivity disorder, BMI: body mass index, WCadjBMI(ns): Waist circumference adjusted for body mass index (in non-smokers), RBC: red blood cell, Hb: Hemoglobin, SIA: Sulfasalazine-induced agranulocytosis, IMBadjBS: Itch intensity from mosquito bite adjusted by bite size, UTI: Urinary tract infection frequency, PUM: Perceived unattractiveness to mosquitoes, TIBC: total iron binding capacity**.**
